# Seed-Derived Synthetic Microbial Communities (SynComs) from *Medicago* Wild Relatives Modulate Early Plant Microbiome Assembly and Phenotypic Traits in Lucerne (*Medicago sativa* L.)

**DOI:** 10.3390/microorganisms13092114

**Published:** 2025-09-10

**Authors:** Shenali Subodha Herath Dissanayakalage, Jatinder Kaur, Tongda Li, Adam M. Dimech, Timothy I. Sawbridge

**Affiliations:** 1 Department of Energy, Environment and Climate Action, Agriculture Victoria, AgriBio, Centre for AgriBioscience, Bundoora, VIC 3083, Australia; jatinder.kaur@agriculture.vic.gov.au (J.K.); tongda.li@agriculture.vic.gov.au (T.L.); adam.dimech@agriculture.vic.gov.au (A.M.D.); tim.sawbridge@agriculture.vic.gov.au (T.I.S.); 2 School of Applied Systems Biology, La Trobe University, Bundoora, VIC 3083, Australia

**Keywords:** synthetic bacterial communities, SynComs, *Medicago sativa*, plant–microbe interactions, plant growth promotion, microbiome, high-throughput phenotyping, 16S rRNA gene sequencing, drought stress, plant phenomics

## Abstract

Seed-associated microbiomes represent an underexplored frontier in synthetic community (SynCom) design, particularly in forage legumes such as lucerne (*Medicago sativa* L.), where early microbial assembly can shape plant development. Crop wild relatives (CWRs) harbour more diverse seed microbiomes and may contain microbes with greater functional potential than domesticated lucerne. To test this, SynComs were constructed from seed-borne bacteria isolated from *M. laciniata* (drought-resilient) and *M. littoralis* (salt-tolerant). Two three-strain SynComs were assembled from taxa consistently shared across lucerne and its CWRs, and a third six-strain ‘Mix’ SynCom combined both sets. The aim of this study was to assess whether these SynComs exert phenotypic effects on lucerne growth when used as seed inocula alongside the native microbiome during early development and later vegetative stages under well-watered and drought conditions. Inoculation enhanced germination and early growth, with the Mix SynCom producing the strongest gains. Microbiome profiling at 24 days revealed treatment-specific restructuring, with enrichment of beneficial taxa and microbial coalescence. While early-stage benefits diminished at later stages, and drought ultimately reduced biomass across all treatments, the findings demonstrate that CWR-derived SynComs can enhance lucerne establishment and early growth while restructuring host microbiomes, providing a framework for seed-applied microbial solutions in sustainable agriculture.

## 1. Introduction

Plants interact with diverse microbial communities that influence nutrient acquisition, stress adaptation, and immune regulation, forming intricate ecological networks. These interactions are governed by the host genotype (GP), microbiome composition (GM), and environmental conditions (E)—a framework often described as plant health = GP × GM × E [1,2,3]. More recently, these relationships have been conceptualised within the broader context of plant holobiont dynamics, which emphasises the host and its associated microbiota as an integrated unit of selection and adaptation [4]. Although extensive research has focused on the rhizosphere and phyllosphere microbiomes, the seed microbiome remains an understudied yet crucial determinant of plant health, influencing early microbial assembly and plant development [5,6,7,8]. However, its potential for microbiome engineering remains largely untapped, partly due to the complexity of plant–microbe and microbe–microbe interactions [9].

Recent advances in microbiome research have enabled the development of synthetic microbial communities (SynComs), providing a controlled and reproducible approach for studying plant–microbe interactions and manipulating microbiomes with precision [10,11]. SynCom applications across a range of crops have demonstrated their potential to enhance plant fitness, microbial stability, and soil health. For instance, in cotton (*Gossypium hirsutum*), seed-applied SynComs improved germination, biomass accumulation, and yield, while modulating beneficial rhizosphere taxa [12]. In walnuts (*Juglans regia*), a three-strain *Bacillus*-based SynCom (*B. safensis* 5-49, *B. halotolerans* 6-30, and *B. stratosphericus* 5-54) significantly enhanced seedling biomass and nutrient mobilisation through microbial interactions [13]. Similarly, in sorghum (*Sorghum bicolor* L.), SynCom studies have identified key bacterial taxa that influence root system development and drought adaptation [14]. While these studies demonstrate the potential of SynComs to support plant growth, they also imply a broader functional role in shaping microbial community assembly—an ecological dimension that remains incompletely characterised.

Despite these advances, most SynComs to date have been derived from root-associated microbiota, with relatively little attention given to seed-origin microbial consortia. A recent study demonstrated that a seed-derived SynCom in radish (*Raphanus sativus*) effectively shaped both seed and seedling microbiota, reinforcing its potential to guide early-stage microbial assembly [15]. However, this approach remains underutilised in lucerne (*Medicago sativa* L.), where research has predominantly focused on root- and nodule-derived consortia. This emphasis reflects the long-standing recognition of root nodulation and rhizobial symbiosis as central to the lucerne productivity [16,17], together with the practical accessibility of root-associated microbes compared with those carried internally by seeds. For instance, a four-strain, nodule-derived SynCom in lucerne was shown to alleviate heavy metal, salinity, drought, and temperature stresses by enhancing antioxidant activity, photosynthesis, biomass, nodulation, and nitrogen fixation [18]. Despite such demonstrated benefits, the potential of seed-derived consortia in lucerne remains largely untested.

Lucerne or alfalfa is a globally significant forage legume, critical to the dairy industry owing to its high nutritional value, digestibility, and palatability [19,20]. Its productivity is closely linked to microbial symbiosis, particularly biological nitrogen fixation and other root-associated mutualisms [21,22]. Evidence from other legumes shows that crop wild relatives (CWRs) often harbour distinct seed-associated microbiota compared with domesticated counterparts, reflecting their ecological adaptations [23]. In *Medicago*, wild taxa such as *M. laciniata* and *M. littoralis* represent reservoirs of beneficial microbes that may contribute to microbiome stability and adaptive resilience. However, their translational potential remains largely unexplored [24].

To address this knowledge gap, we developed an ecologically informed, reductionist SynCom design strategy that diverges from conventional trait-based approaches. SynComs were constructed exclusively from seed-associated bacterial isolates obtained from *M. laciniata* and *M. littoralis*. Constituent taxa were selected based on consistent co-occurrence across wild relatives and lucerne, as well as high genomic similarity to lucerne-associated strains. This rational, compatibility-informed approach contrasts with empirical, combinatorial methods and proposes a new paradigm for SynCom development—one that prioritises ecological fit and evolutionary continuity.

We hypothesised that CWR-derived microbes would enhance lucerne microbiome stability by selectively enriching functionally compatible taxa, thereby influencing seedling establishment and plant growth under both well-watered and drought conditions. Each SynCom, comprising three bacterial species per CWR, was applied via seed inoculation, and plant responses were evaluated across multiple developmental stages. A comprehensive experimental framework integrating morphometric analysis, biomass assessment, 16S rRNA gene sequencing, and high-throughput (HTP) phenotyping was employed to capture both plant phenotypic responses and microbial community shifts. Collectively, these approaches allowed us to assess whether CWR-derived SynComs function not merely as biofertilisers, but as modulators of microbial assembly and plant–microbe dynamics. By linking phenotypic outcomes to microbiome composition, this study establishes a proof-of-concept for seed-derived SynComs in lucerne, and highlights how ecological design principles can inform future microbiome-based strategies in forage crop improvement.

## 2. Materials and Methods

### 2.1. Selection of Bacterial Strains and SynCom Inoculum Preparation

Six bacterial strains were selected from a previously constructed library of 530 seed-derived bacterial isolates from lucerne and its CWRs (Table 1). The library was established through culture-based isolation, and 315 isolates were taxonomically classified using 16S rRNA Sanger sequencing. A subset of 34 representative isolates underwent whole-genome sequencing to investigate their comparative genomic relationships and potential functional roles in lucerne under commercial settings. Average Nucleotide Identity (ANI)-based genomic analysis identified taxa that were consistently present across both domesticated lucerne and its CWRs, *M. laciniata* and *M. littoralis* [25]. These CWRs were selected due to their contrasting ecological adaptations, with *M. laciniata* exhibiting strong drought resilience [26,27] and *M. littoralis* demonstrating moderate salt tolerance [28]. Rather than undertaking a direct comparison between isolates from lucerne and those from its wild relatives, the present study focused on microbial taxa naturally shared by both groups. By introducing CWR-derived strains of these shared taxa into lucerne, we sought to evaluate whether microbes originating from stress-adapted wild relatives could differentially influence lucerne growth and microbiome assembly, and whether such strains may confer advantages not evident in domesticated seed-associated microbiota. Although functional assays were not performed in this study, the selected taxa are well-documented for plant-associated traits. *Pantoea* spp. have been reported to solubilise phosphate, produce siderophores, and regulate phytohormones, while *Pseudomonas* spp. (including *P. graminis*) are recognised for ACC deaminase activity, antifungal metabolite production, and stress mitigation [29,30,31]. These established roles highlight their ecological relevance as SynCom members, even in the absence of strain-specific characterisation.

**Figure 1:**
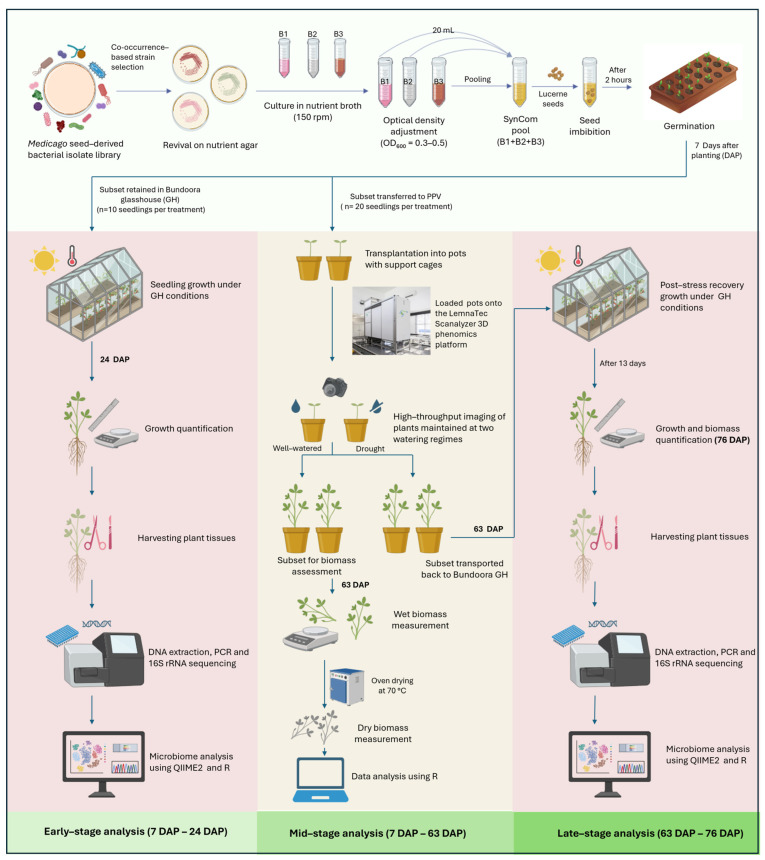
Schematic overview of the synthetic community (SynCom) experimental workflow. Bacterial isolates from *Medicago* crop wild relatives (CWRs) were cultured and pooled into SynComs based on optical density. Lucerne seeds were inoculated via seed imbibition and germinated for 7 days. A subset of seedlings (*n* = 10 seedlings per treatment) was retained in the glasshouse for early-stage phenotyping and microbiome analysis at 24 days after planting (DAP), while the remainder were transferred to the Plant Phenomics Victoria (PPV) facility and imaged under two watering regimes until 63 DAP. At 63 DAP, subsets were harvested for biomass measurement (*n* = 5 seedlings per treatment) or returned to the glasshouse for recovery (*n* = 3 seedlings per treatment). Late-stage growth and microbiome analysis were conducted at 76 DAP (exploratory due to limited replication). Microbiome profiling was based on 16S rRNA sequencing and analysed using QIIME2 and R. Icons adapted from BioRender.com; figure assembled in Microsoft PowerPoint.

To construct SynComs, three formulations were developed: (i) a Laciniata (LA) consortium, comprising three bacterial strains from *M. laciniata*; (ii) a Littoralis (LT) consortium, consisting of three bacterial strains from *M. littoralis*; and (iii) a Mix consortium, combining all six bacterial strains from LA and LT consortia (Supplementary Table S1). To assemble these SynComs, cryopreserved bacterial isolates were revived on nutrient agar and subsequently cultured in 40 mL of nutrient broth (NB) at 28 ± 1 °C with constant agitation at 150 revolutions per minute (rpm) in a shaker incubator. Following overnight incubation, bacterial cell densities were adjusted to an optical density at 600 nm (OD_600_) of approximately 0.3–0.5, ensuring uniform cell density across strains. Equal volumes (20 mL) of each adjusted culture were pooled under sterile conditions to assemble the final SynCom inoculums (Figure 1).

#### Cultivar Selection and Germination

Lucerne cultivar ‘Aurora’ (Australian Wheatgrass Company, New South Wales, Australia) was selected for *in planta* evaluation of SynCom treatments. The seeds were not chemically treated or coated by the supplier. Seeds were surface rinsed four times with autoclaved reverse osmosis (RO) water; during the final rinse, seeds were incubated for 4 min at room temperature to facilitate the removal of contaminants and loosely associated bacteria from the seed coat. A 100 µL aliquot of the final wash was plated onto Reasoner’s 2A (R2A; Oxoid, South Australia, Australia or Amyl Media, Victoria, Australia) and incubated at room temperature for seven days, confirming the absence of culturable bacteria. As the aim of this study was to evaluate SynCom performance in the context of the native seed microbiome, we did not attempt to remove all epiphytic or endophytic bacteria, thereby maintaining ecological relevance to field application. Seeds were then imbibed in each bacterial consortium solution for 2 h with constant agitation at 150 rpm prior to germination. Control seeds underwent identical treatment but were imbibed in sterile NB without any bacterial inoculants. Germination rates were manually recorded at 7 days after planting (DAP).

### 2.2. Overview of the Conventional Phenotyping and HTP Imaging of the SynCom Effects Across Developmental Stages

**Figure 2:**
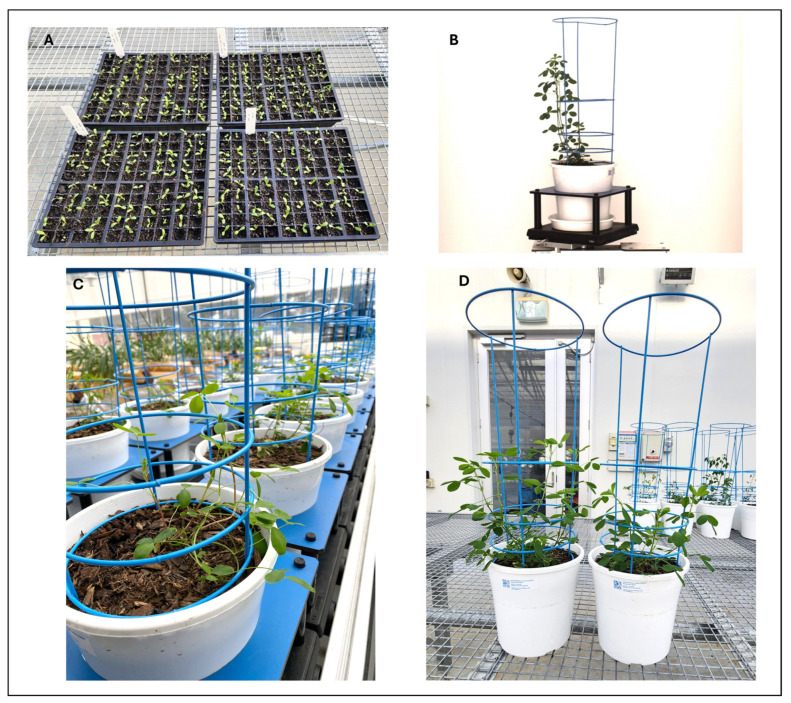
(**A**) Seed germination of lucerne cultivar ‘Aurora’ in 100-cell trays at 7 DAP in the glasshouse at Bundoora, Victoria, Australia. (**B**) A typical RGB (Red–Green–Blue) image of a lucerne plant captured by LemnaTec Scanalyser 3D high-throughput phenotyping platform at PPV, Horsham, Victoria, Australia. (**C**) Plants growing in the PPV facility at 41 DAP under defined watering regimes. (**D**) Representative plants at 63 DAP, immediately prior to harvesting for destructive biomass measurements. Each treatment group consisted of 20 replicates, evenly divided between two watering conditions: 10 replicates were maintained under well-watered conditions (80% soil gravimetric water content, SGWC), while the remaining 10 were subjected to drought conditions.

To assess stage-specific effects of SynCom treatments, all plants were germinated as a single batch in a controlled glasshouse facility at the Centre for AgriBioscience in Bundoora, Victoria, Australia (37°41′51″ S, 145°04′38″ E). At 7 DAP, a subset of seedlings (*n* = 20 per treatment) was transferred to the Plant Phenomics Victoria (PPV) facility in Horsham, Victoria, Australia (36°43′13.8″ S, 142°10′25.3″ E), where they were transplanted into pots and maintained for HTP imaging until 63 DAP (Figure 2A–D). The remaining seedlings continued to grow in the Bundoora glasshouse, where early-stage phenotyping was conducted at 24 DAP. Following mid-stage phenotyping (63 DAP) in the PPV facility under both well-watered and drought conditions, a subset of plants (*n* = 3 per treatment) was transported back to the Bundoora glasshouse and maintained under standard glasshouse conditions until 76 DAP, when post-stress recovery measurements were collected.

#### 2.2.1. Early Seedling Growth Under Glasshouse Conditions

SynCom-inoculated and Control treatment seeds were germinated and grown in 100-cell seedling trays (cell size: 30 mm × 30 mm × 50 mm) filled with standard potting mix (Van Schaik’s Bio Gro Pty. Ltd., Dandenong South, Victoria, Australia; composition details in Supplementary Section S1) for 24 days. The potting substrate was amended with extra-coarse vermiculite, Macracote Blue Coloniser Plus (Sunpalm Australia Pty. Ltd., Wangara, Western Australia, Australia), nitrogen fertiliser (Sirflo Plus N, 39.5% N), water-retaining crystals (Seasol, Boronia, Victoria, Australia), and garden lime. A total of 40 seedlings (10 replicates per treatment) were maintained in the glasshouse under controlled conditions: 22 °C daytime temperature (6:00 to 20:00), with artificial lighting activated when ambient light intensity dropped below 170 W/m^2^, and nighttime temperatures of 14 °C, with relative humidity maintained at 50–60%.

#### 2.2.2. Controlled Environments and Phenotyping Setup at PPV Facility, Horsham

At 7 DAP, seedlings were transported in their original trays to the PPV facility in Horsham, where they were transplanted into 200 mm diameter × 200 mm height white plastic pots (catalogue P200E04, Garden City Planters Pty. Ltd., Dandenong South, Victoria, Australia) filled with the same potting mix as previously described. To minimise foliage overhang and ensure uninterrupted imaging accuracy, a powder-coated blue (Reichs-Ausschuss für Lieferbedingungen [RAL] 5012 ‘Light Blue’) resin wire cage was installed in each pot at the time of transplantation. Each pot was placed in an individual carrier equipped with a radio frequency identification (RFID) chip and loaded onto the LemnaTec Scanalyser 3D (LemnaTec GmbH, Aachen, Germany) HTP phenomics platform to evaluate SynCom effects on lucerne. The seedlings were grown in a climate-controlled glasshouse at 23 °C from 7:00 to 20:00 and 15 °C from 20:00 to 7:00 without supplemental light. The glasshouse was clad in double-walled plexiglass (poly(methyl methacrylate), PMMA) sheeting, which permitted transmission of the full spectrum of sunlight.

Each treatment group consisted of 20 replicates, evenly divided between two watering conditions: 10 replicates were maintained under well-watered conditions (80% soil gravimetric water content, SGWC), while the remaining 10 were subjected to drought conditions (40% SGWC). To ensure uniform establishment post-transplantation, all plants were initially irrigated to 80% SGWC twice daily for the first week (see Supplementary Section S2 for SGWC details). This acclimation phase was implemented to support root system establishment and minimise transplant shock, as sudden exposure to severe water stress can negatively impact seedling survival and early growth. Following the acclimation phase, watering in the drought treatment was gradually reduced to 40% SGWC, while plants in the well-watered treatment were maintained at 80% SGWC. Watering was applied by the LemnaTec Scanalyser, which used pot weight to determine the amount of water to be applied for each treatment on a daily basis.

#### 2.2.3. Early-Stage Phenotyping at Glasshouse, Bundoora (24 DAP)

At 24 DAP, seedlings were harvested (*n* = 10 per treatment) for shoot and root measurements, and tissue samples were collected for 16S rRNA gene sequencing.

#### 2.2.4. Mid-Stage Phenotyping at PPV, Horsham (63 DAP)

Each treatment–watering condition combination consisted of ten biological replicates, from which five plants (*n* = 5) were randomly selected for biomass assessment. Aerial tissues were harvested and immediately weighed to record fresh/wet biomass. Samples were subsequently oven-dried at 70 °C for three days, after which dry biomass was recorded.

#### 2.2.5. Late-Stage Phenotyping at Glasshouse, Bundoora (76 DAP)

Following mid-stage phenotyping at 63 DAP, three plants per treatment (*n* = 3) were transferred to the Bundoora glasshouse and maintained under standard watering conditions for an additional 13 days. Root and shoot biomass and length were measured, and tissue samples were collected for 16S rRNA gene sequencing. However, sequencing data from this time point were excluded from downstream analysis due to limited replication and variable taxonomic profiles.

### 2.3. HTP Image Analysis

Plants were imaged twice weekly on the LemnaTec Scanalyser 3D automated phenomics platform using visible-spectrum (red–green–blue, RGB) cameras under controlled lighting. Images were captured from above (top view, TV) and three side angles (0°, 120°, 240°) and stored in a PostgreSQL database. A customised PlantCV [32] pipeline (v4.5.1, OpenCV v4.10.0.84, Python v3.12.10, CentOS v7.9) was developed and run on the Biosciences Advanced Scientific Computer (BASC) cluster to process images. The pipeline included background segmentation, removal of support structures, and isolation of plant regions from both side- and top-view images. Digital volume, derived from combined green pixel areas across views, was calculated using V_LemnaTec_ formula [33], and used as a validated proxy for biomass. Full details of image processing workflow, including colour space conversions, thresholds, and morphological operations, are provided in the Supplementary Section S3.

### 2.4. DNA Extraction, 16S rRNA Amplicon Library Preparation and Sequencing

Root and shoot samples collected at 24 DAP and 76 DAP were used for DNA extraction and 16S rRNA amplicon library preparation, which was performed using a modified Qiagen^®^ MagAttract^®^ protocol. The sequencing was conducted on an Illumina MiSeq platform (2 × 300 bp). Detailed library preparation and sequencing protocols are provided in Supplementary Section S4.

### 2.5. Data Analysis

#### 2.5.1. Statistical Analysis for Conventional Phenotyping Measurements

All statistical analysis and visualisations were conducted using R (version 4.4.2, R Core Team, 2024) with tidyverse, car, FSA, emmeans, and multcompView packages. The datasets from different phenotyping timepoints were reshaped to a long format using pivot_longer function (tidyr), with treatment labels harmonised using recode. Descriptive statistics including means, standard deviations (SD), standard errors (SE), and fold changes were calculated for each treatment and condition. Assumption testing was performed using the Shapiro–Wilk test for normality and Levene’s test for homogeneity of variances. Where assumptions were met, one-way analysis of variance (ANOVA) was performed, followed by Tukey’s Honest Significant Difference (HSD) test (Tukey HSD) for pairwise comparisons. For data that did not satisfy these assumptions, a Kruskal–Wallis test was applied, with pairwise Wilcoxon rank-sum tests used for post hoc comparisons with Bonferroni correction. Fold changes in mean values were calculated to compare the treatment effects across time points, while statistical significance was assessed using ANOVA or Kruskal–Wallis tests, as appropriate. A threshold of *p* < 0.05 was used to determine statistical significance.

#### 2.5.2. Validation of HTP-Derived Biomass Estimates

To evaluate the relationship between digital volume estimates from HTP and conventional biomass measurements, wet aerial biomass at 63 DAP was compared against pixel-derived digital volume data. Both datasets were analysed in their original units to retain scale integrity. A linear regression model was applied in R (v4.4.2), and model fit was assessed using the coefficient of determination (*R*^2^). An identity line (wet biomass regressed against itself) was included for reference. Data reshaping and preparation were performed using the dplyr and tidyr packages, and visualisations were generated using ggplot2.

#### 2.5.3. Microbiome Data Processing and Statistical Analysis

Microbiome analysis was conducted using a combination of QIIME2 (Quantitative Insights Into Microbial Ecology 2; v2021.4) and R to process, filter, and analyse the 16S rRNA sequencing data. Raw paired-end reads were initially merged and error-corrected using PEAR (Paired-End reAd mergeR; v0.9.10) with default parameters [34]. The merged sequences were imported into QIIME2 using the manifest format as single-read FASTQ files. To remove primer sequences, the q2-Cutadapt plugin was applied with an error rate of 0.2 and the following parameters: error rate-0.2, flags; adapter-wildcards, read-wildcards, and discard-untrimmed. Demultiplexed raw sequences were processed, where low-quality reads were trimmed, and sequences were denoised using DADA2 (Divisive Amplicon Denoising Algorithm 2) [35] to infer amplicon sequence variants (ASVs) while simultaneously filtering out chimeric sequences. Taxonomic classification was performed using a Naïve Bayes classifier trained on the SILVA 138 database at a 99% identity threshold [36]. To ensure accurate representation of microbial communities, cytoplasmic contaminants such as chloroplast, mitochondria, or eukaryotic sequences were removed from the dataset. The filtered ASV table, taxonomy assignments, and associated metadata were then imported into R for downstream analyses using the phyloseq, microbiome, vegan, and indicspecies packages [37,38].

Alpha diversity metrics [39], including Shannon, Simpson, Chao1, and Observed ASVs, were calculated to assess within-sample microbial diversity using the phyloseq and vegan packages. Observed ASVs and Chao1 reflect species richness, while Shannon and Simpson indices incorporate both richness and evenness. Differences in alpha diversity between treatments were statistically tested using Kruskal–Wallis tests, followed by pairwise Wilcoxon rank-sum tests with Benjamini–Hochberg correction for multiple comparisons [40]. Core microbiome analysis was performed in R using the phyloseq package, applying a prevalence threshold of 50% (i.e., taxa present in at least 50% of samples within a group) and an abundance detection threshold of 0.001. Indicator species analysis was performed using the indicspecies package in R to identify microbial taxa significantly associated with specific SynCom treatments, with statistical significance assessed using 999 permutations. This method was selected as it was well-established for detecting treatment-specific microbial signatures in SynCom studies [12]. Data visualisation and statistical analyses were conducted in R, with ggplot2 and ggpubr used for diversity plots and statistical comparisons. Core microbiome overlaps were visualised using the VennDiagram package. Relative abundance transformations were applied where necessary to ensure valid comparisons across samples.

## 3. Results

### 3.1. Microbiome Dynamics at 24 DAP

#### 3.1.1. Microbiome Sequencing and Data Processing

A total of 43,159,116 raw reads were generated across all samples (Supplementary Table S2). After quality filtering, 10,461,259 high-quality reads were retained. Removal of plant-derived sequences resulted in 9,521,338 non-plant microbial reads. Prior to rarefaction, sequencing depth varied substantially across samples, from 0 to 504,125 reads per sample, with a median 28,893. To ensure comparability, samples were rarefied to 20,000 reads, retaining 47 samples for downstream analyses. The resulting dataset comprised 940,000 sequences and 3299 ASVs, capturing representative microbial diversity for robust analysis.

#### 3.1.2. Microbial Diversity Analysis

**Figure 3:**
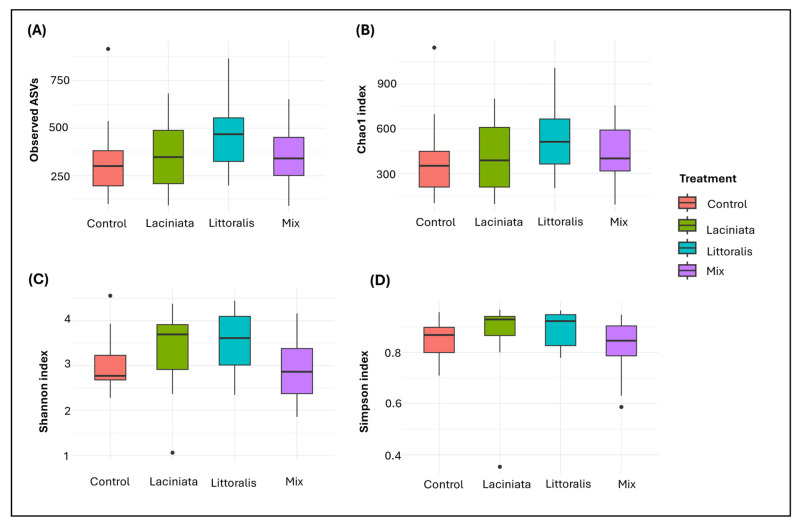
Alpha diversity of microbial communities associated with lucerne plant tissue samples at 24 DAP under different SynCom treatments. Boxplots show (**A**) Observed ASVs, (**B**) Chao1 richness, (**C**) Shannon diversity index, and (**D**) Simpson index for four treatment groups: Control, Laciniata, Littoralis, and Mix. Diversity metrics were calculated from rarefied amplicon sequence variants (ASV) tables. Boxes represent interquartile range (IQR), with the horizontal line indicating median; whiskers extend to 1.5 × IQR. Individual points represent potential outliers.

Alpha diversity was assessed using Observed ASVs, Chao1, and Shannon and Simpson indices to evaluate microbial richness and evenness across treatments (*n* = 10 per treatment group). While statistical analysis revealed no significant differences in alpha diversity among treatments (Observed ASVs: *p* = 0.280, Chao1: *p* = 0.424, Shannon: *p* = 0.197, Simpson: *p* = 0.196), descriptive patterns/trends observed in richness and diversity metrics suggest potential ecological shifts in microbial community composition among SynCom-inoculated samples compared to Control. LT exhibited the highest values for both richness and diversity indices, followed by LA and Mix, with the relative ranking differing slightly across indices. The Control consistently showed the lowest values across all metrics (Figure 3A–D).

#### 3.1.3. Core Microbiome Analysis

**Figure 4:**
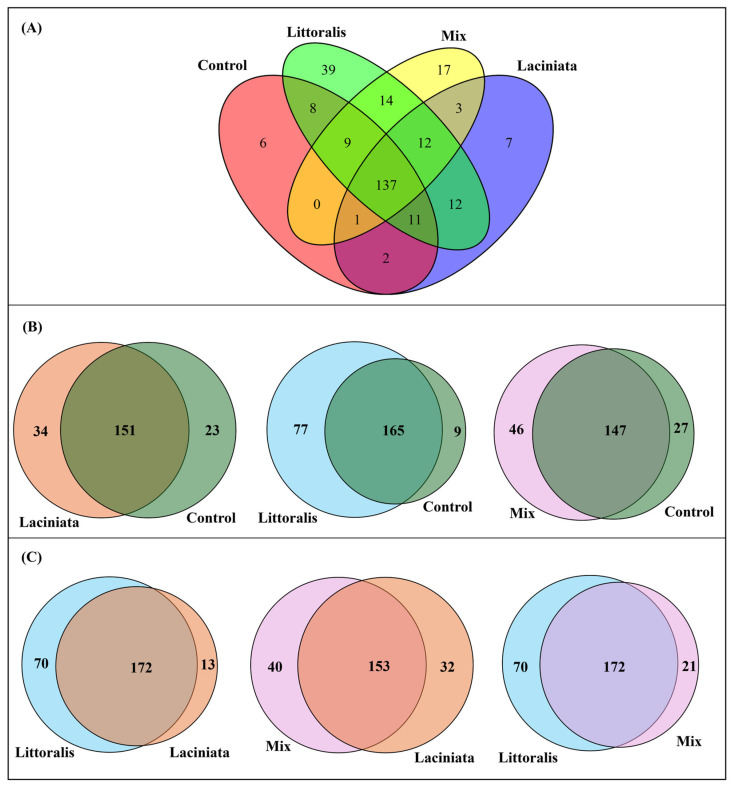
Core microbial ASVs shared across treatments at 24 DAP. (**A**) Four-way Venn diagram showing the number of core ASVs shared among Control, Laciniata, Littoralis, and Mix treatments. Core ASVs were defined as ASVs present in ≥50% of samples within each group at a detection threshold of 0.001. (**B**) Pairwise Venn diagram comparing core ASVs between each SynCom treatment and Control: Laciniata vs. Control (**left**), Littoralis vs. Control (**middle**), and Mix vs. Control (**right**). (**C**) Pairwise comparisons of core ASVs among SynCom treatments: Littoralis vs. Laciniata (**left**), Mix vs. Laciniata (**middle**), and Littoralis vs. Mix (**right**).

To further characterise microbial community composition, core taxa were identified and compared between the Control and SynCom-treated groups, as well as among SynCom treatments. Core taxa were defined as ASVs present in ≥50% of samples within each group at a detection threshold of 0.001. A total of 137 ASVs were consistently shared across all treatments, including the Control. These belonged to the classes Alphaproteobacteria (*Allorhizobium–Neorhizobium–Pararhizobium–Rhizobium* group [ANPR], *Azospirillum*, *Bradyrhizobium*, *Novosphingobium*, *Pseudolabrys*, *Sphingomonas*), Gammaproteobacteria (*Massilia*, *Pantoea*, *Pseudomonas*), Actinobacteria (*Streptomyces*, *Cellulomonas*), and additional members from Bacteroidia, Bacilli, Bdellovibrionia, Holophagae, Myxococcia, Polyangia, Saccharimonadia, Thermoleophila, and Verrucomicrobiae (Figure 4A; a complete taxonomic list is available in Supplementary Table S3). Taxonomic annotations for ASVs were assigned using standard classification algorithms against the SILVA database (v138) and reflect the most probable phylogenetic affiliations based on available reference sequences. These labels will be used throughout the discussion to aid in interpreting potential ecological roles, while acknowledging the limitations of inference based solely on taxonomic assignment.

The total number of core ASVs detected was 208 in LT, 206 in LA, 199 in Mix, and 208 in the Control group. Pairwise comparisons with the Control revealed a substantial overlap in core taxa, with shared ASVs ranging from 65.7% to 72.6% (Table 2). The LA treatment showed the highest overlap with the Control (72.6%), followed by Mix (66.8%) and LT (65.7%) (Figure 4B). LT also contained a larger proportion of unique ASVs (30.7%) compared to Control, a difference that was statistically significant (Fisher’s Exact Test: *p* = 3.02 × 10^−12^; Chi-square: *p* = 8.18 × 10^−11^), indicating the introduction or enrichment of distinct taxa in this treatment. The Mix treatment also harboured 20.9% unique ASVs, though this difference was marginally non-significant (*p* = 0.0502).

Comparisons among SynCom treatments revealed moderate overlap in core taxa (65.4–68.0%), with LT again showing the highest proportion of unique ASVs—27.5% compared to LA and 26.6% compared to Mix (Table 2; Figure 4C). These differences were statistically significant (Fisher’s Exact Test: LT vs. LA: *p* = 6.14 × 10^−9^; LT vs. Mix: *p* = 3.09 × 10^−6^), indicating treatment-specific microbial associations in the LT group. In contrast, Mix treatment exhibited the lowest proportion of unique core taxa (8.0%), consistent with its previously observed lower alpha diversity.

A total of 149 ASVs, representing Alphaproteobacteria, Gammaproteobacteria, Actinobacteria, Bdellovibrionia, Verrucomicrobiae, Holophagae, Myxococcia, Polyangia, Saccharimonadia, and Bacilli, were shared across all three SynCom treatments (complete taxonomic list is provided in Supplementary Table S4). Additionally, 12 ASVs were uniquely detected in SynCom treatments but absent in the Control. These included taxa from *Novosphingobium*, *Pseudolabrys*, *Bdellovibrio*, Solirubrobacterales_67–14, Microscillaceae (uncultured), Rhizobiacea (unclassified), LWQ8, Pedosphaeraceae (uncultured), and Opitutaceae (uncultured) (see Supplementary Table S5).

#### 3.1.4. Indicator Taxa Analysis

**Figure 5:**
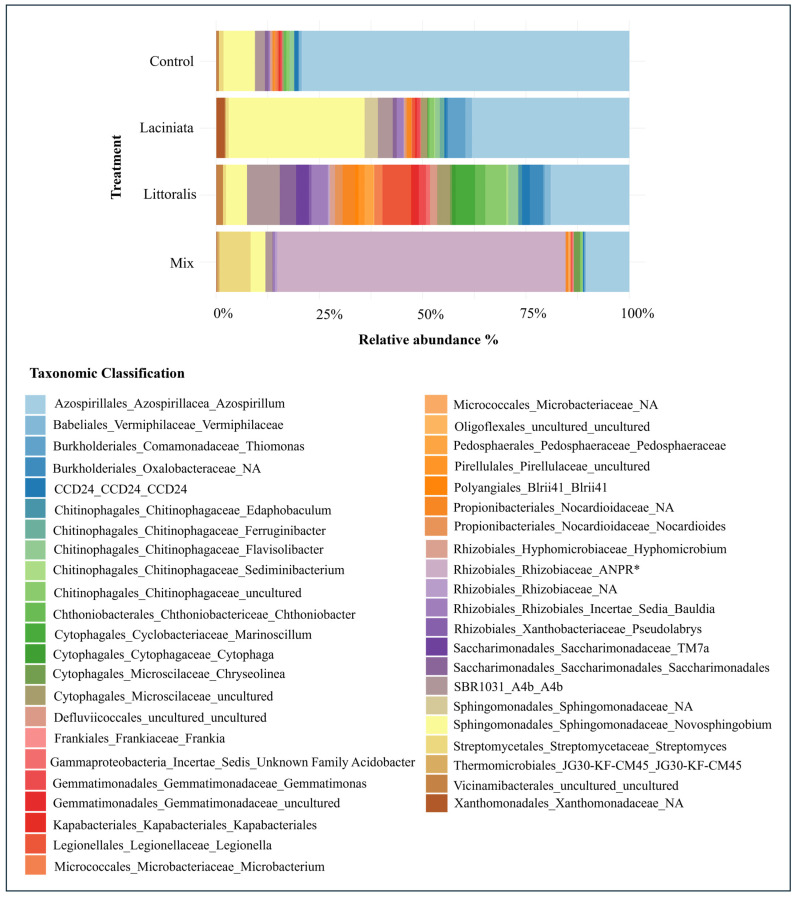
Relative abundance of indicator bacterial taxa identified across SynCom treatments at 24 DAP. Indicator taxa were determined using indicator species analysis (indicspecies package in R, 999 permutations), based on their specificity and fidelity to each treatment. Taxa were reported at the lowest available taxonomic level, with labels reflecting hierarchical annotation (Order–Family–Genus). A prevalence threshold of ≥50% and a relative abundance threshold of ≥0.001 were applied. Bars represent the relative abundance of each indicator taxon in lucerne across replicates (*n* = 10 per treatment) for Control, Laciniata, Littoralis, and Mix treatments.

Indicator taxa analysis was performed using the indicspecies package to identify microbial groups significantly associated with specific SynCom treatments and abundant within communities. Rather than broadly profiling differential abundance across the community, this approach focused on detecting microbial taxa with high fidelity and specificity to individual treatments. A total of 27 bacterial orders were detected across all samples (Figure 5). The microbial community composition revealed distinct treatment-specific signatures, with certain orders significantly associated with particular SynCom inoculations.

Importantly, indicator taxa identified here are not the inoculated SynCom members themselves, but native or coalesced taxa whose abundance was significantly associated with specific treatments. This distinction highlights the capacity of SynComs to modulate broader microbiome assembly without necessarily dominating the community.

The Control group was strongly dominated by Azospirillales, comprising over 75% of the total relative abundance. In contrast, the LA treatment exhibited a notable shift, with enrichment of Sphingomonadales (36.19%) and Burkholderiales (4.56%) compared to the Control. The LT treatment displayed the greatest diversity of indicator taxa among all treatment groups, with Azospirillales (19.01%), Cytophagales (9.20%), and Chitinophagales (8.82%) emerging as dominant contributors. This broader distribution of taxa suggests a more extensive microbial restructuring in response to LT inoculation. The Mix treatment presented a distinct microbial profile, dominated by Rhizobiales, which constituted 71.01% of the total community composition (see Supplementary Table S6 for the complete list of indicator taxa and their relative abundances).

### 3.2. Overview of Conventional Phenotyping Measurements

Conventional (destructive) phenotyping was performed to assess the effects of SynCom treatments on lucerne growth and biomass accumulation across different developmental stages and watering regimes. Growth parameters were measured at three time points (24, 63, and 76 DAP) in lucerne cultivar ‘Aurora’. At 24 DAP, root and shoot lengths were measured under standard glasshouse conditions, capturing seedling establishment and early growth responses. At 63 DAP, wet and dry aerial biomass measurements were recorded at the PPV facility under well-watered and drought conditions to assess mid-stage treatment responses to water availability. At 76 DAP, following re-establishment in the glasshouse, root and shoot length and biomass were measured to evaluate post-stress growth recovery.

#### 3.2.1. Evaluating the Impact of SynCom Treatments on Germination and Early-Stage Plant Growth

**Figure 6:**
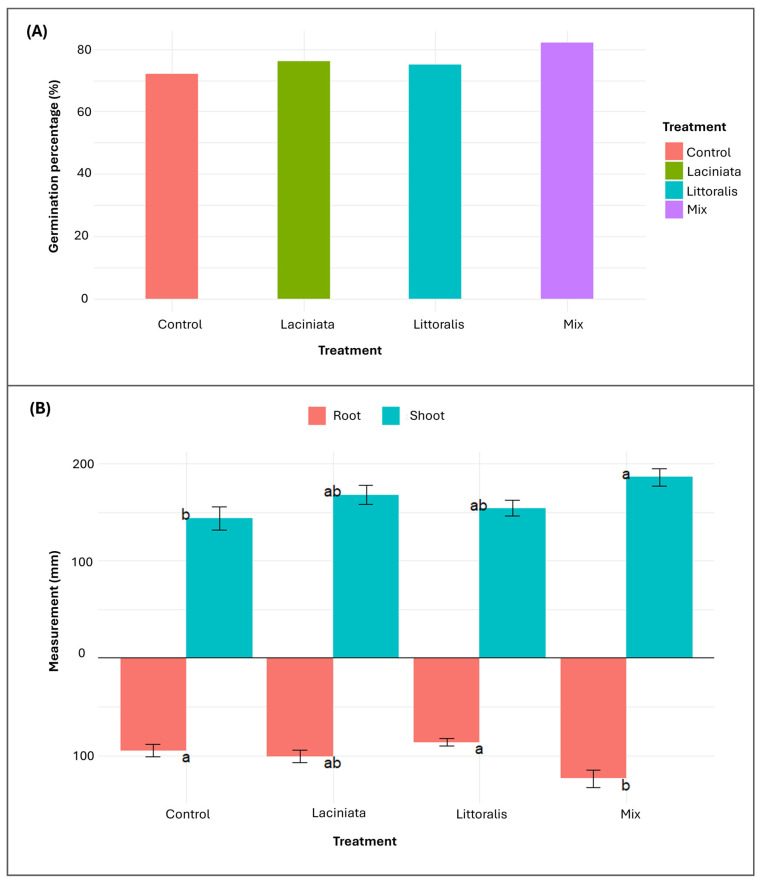
Germination percentage and early-stage growth responses of lucerne seedlings under SynCom treatments. (**A**) Germination percentage (%) at 7 DAP for lucerne seeds treated with three SynComs, Laciniata, Littoralis, and Mix, compared to an uninoculated Control (*n* =100 seeds per treatment). (**B**) Root and shoot lengths at 24 DAP across treatments. Bars heights indicate mean values (*n* = 10 per treatment) ± standard error (SE). A mirrored bar plot was used to display root length (below *x*-axis) and shoot length (above). Different letters denote statistically significant differences among treatments (Tukey’s HSD test, *p* < 0.05).

The Mix treatment exhibited the highest germination rate (82%), followed by LA (76%), LT (75%), and Control (72%) (Figure 6A). As differences were minimal and not central to the study’s primary objective, no statistical analysis was performed. At 24 DAP, root and shoot lengths were measured to assess treatment effects on early growth (Figure 6B). Both datasets met assumptions of normality (Shapiro–Wilk test, *p* > 0.05) and homogeneity of variance (Levene’s test, *p* > 0.05), allowing the use of one-way ANOVA.

The Mix treatment exhibited the greatest mean root (123.7 ± 8.8 mm; 1.3-fold greater than Control) and shoot lengths (186.1 ± 9.2 mm; 1.3-fold greater than Control), significantly exceeding the Control (root: 95 ± 6.6 mm; shoot: 143.5 ± 11.8 mm) (Figure 6B; see Supplementary Table S7 for descriptive statistics summary of 24 DAP measurements). ANOVA revealed statistically significant differences among treatment groups for both root length (*F* = 5.95, *df* = 3, *p* = 0.002) and shoot length (*F* = 3.54, *df* = 3, *p* = 0.024). Post hoc Tukey’s HSD confirmed that Mix treatment differed significantly from the Control in both root (*p* = 0.019) and shoot (*p* = 0.019) measurements. The LA and LT treatments exhibited intermediate growth responses that were not statistically distinct from the Control.

#### 3.2.2. Evaluating Plant Aerial Biomass at 63 DAP Across SynCom Treatments and Watering Conditions

**Figure 7:**
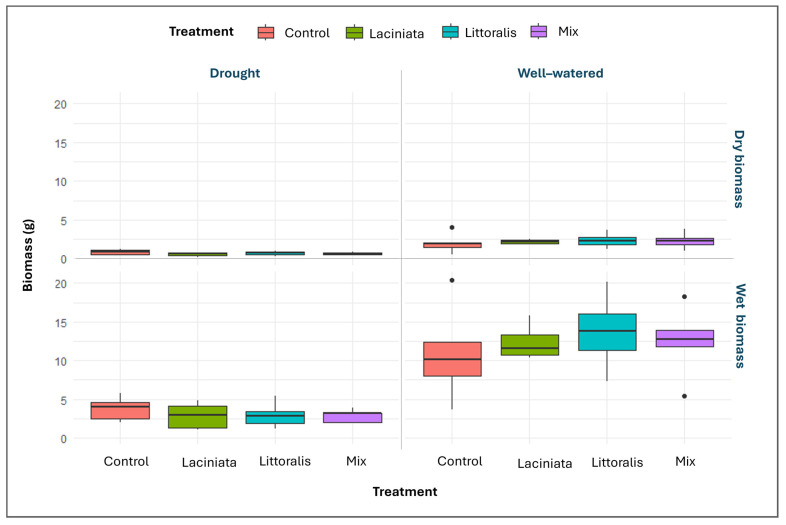
Aerial biomass of lucerne plants at 63 DAP under well-watered and drought conditions across SynCom treatments, Laciniata, Littoralis, and Mix, and uninoculated Control. Boxplots represent dry (**top** panel) and wet (**bottom** panel) shoot biomass for each SynCom treatment and Control. Plants were grown under automated watering regimes in the PPV facility at Horsham, Victoria, Australia. Measurements were conducted under two watering regimes: drought (**left** side) and well-watered (**right** side). Each box represents the interquartile range (IQR), with the horizontal line indicating the median. Whiskers extend to 1.5 × IQR, and individual points represent statistical outliers. Each treatment group includes 10 biological replicates. Biomass values are expressed in grams (g).

As expected, plants grown under well-watered conditions accumulated more aerial biomass than those under drought conditions (Refer to Supplementary Table S8 for descriptive statistics summary of 63 DAP measurements). The data met assumptions of normality (Shapiro–Wilk test, *p* > 0.05) and homogeneity of variance (Levene’s test, *p* > 0.05), allowing one-way ANOVA. No statistically significant differences were observed among treatments for either parameter under either watering condition (*p* > 0.05). However, box plot visualisations (Figure 7) revealed a modest increase in both wet and dry biomass in SynCom-treated plants compared to the Control, particularly under well-watered conditions, with opposite trends seen under drought conditions. Among SynComs, LT showed the greatest mean biomass under optimal watering. Under drought conditions, all SynCom treated plants showed similarly low biomass values, suggesting that inoculation did not enhance growth under water-limited conditions (Supplementary Table S8).

#### 3.2.3. Post-Stress Recovery and Growth Progression Across Timepoints

To explore potential longer-term effects of SynCom treatments under post-stress conditions, a small subset of plants (*n* = 3) were phenotyped at 76 DAP. Due to the limited replication, these observations are exploratory and warrant cautious interpretation. Full data and statistical details are provided in Supplementary Section S5 and Supplementary Table S9.

Among plants previously maintained under well-watered conditions in the PPV facility, LA-treated plants exhibited the highest root length, shoot length, and shoot biomass, while LT-treated plants showed the greatest root biomass. In the previously drought-treated subset, the Mix yielded the greatest root length, LA maintained the highest root biomass, and the Control group exhibited the greatest shoot length and shoot biomass (Supplementary Section S5: Figure S2). Microbiome results are not presented for this time point, as the data were excluded from analysis (see Methods Section 2.2.5).

Fold change analysis between 24 DAP and 76 DAP revealed the largest increase in root length in LT-treated plants, and the greatest increase in shoot length in the Control group. Between 63 DAP and 76 DAP, the LA treatment showed the highest gain in wet aerial biomass, followed by Mix, LT, and Control. Although no statistically significant differences were observed due to the small sample size, these trends provide exploratory insights into treatment-specific recovery dynamics (see Supplementary Section S6 for full analysis).

### 3.3. Overview of HTP Phenotyping Using Image-Based Analysis

HTP imaging was conducted over a 55 day period at the PPV facility, with images captured at 17 time points. To validate the reliability of digital volume as a proxy for aerial biomass, pixel-derived values from HTP imaging were compared with conventional wet biomass measurements obtained at 63 DAP.

#### 3.3.1. Validation of HTP Imaging Against Conventional Biomass Measurements

**Figure 8:**
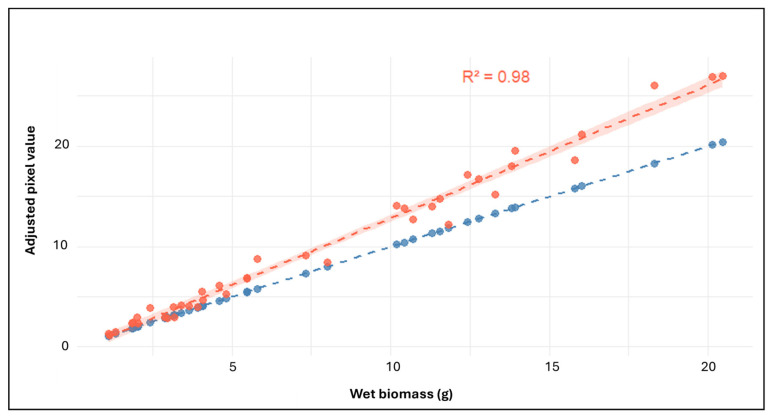
Validation of image-derived biomass estimates against conventional biomass measurements at 63 DAP. Linear regression comparing adjusted pixel values (red) from high-throughput phenotyping (HTP) with conventionally measured wet aerial biomass (g) across all treatments. Pixel-derived estimates show a strong linear relationship with wet biomass (*R*^2^ = 0.98). The red regression line is shown with 95% confidence intervals. A 1:1 reference line (blue) is included for comparison.

A strong positive correlation was observed between HTP-derived digital volume and conventionally measured wet aerial biomass across all treatment groups. Linear regression analysis yielded a *R*^2^ of 0.98, indicating that 98% of the variation in the wet aerial biomass was explained by image-based estimates (Figure 8).

This strong alignment supports the use of pixel-derived measurements as a robust proxy for biomass throughout the imaging period. The consistency in treatment-level trends between adjusted pixel values (calculated by summing pixel data from four camera angles; top view, 0°, 120°, and 240°) and conventional biomass measurements is further illustrated in the comparative bar plot (Supplementary Figure S1).

#### 3.3.2. Early-Phase Imaging Analysis (Day 1–17)

**Figure 9:**
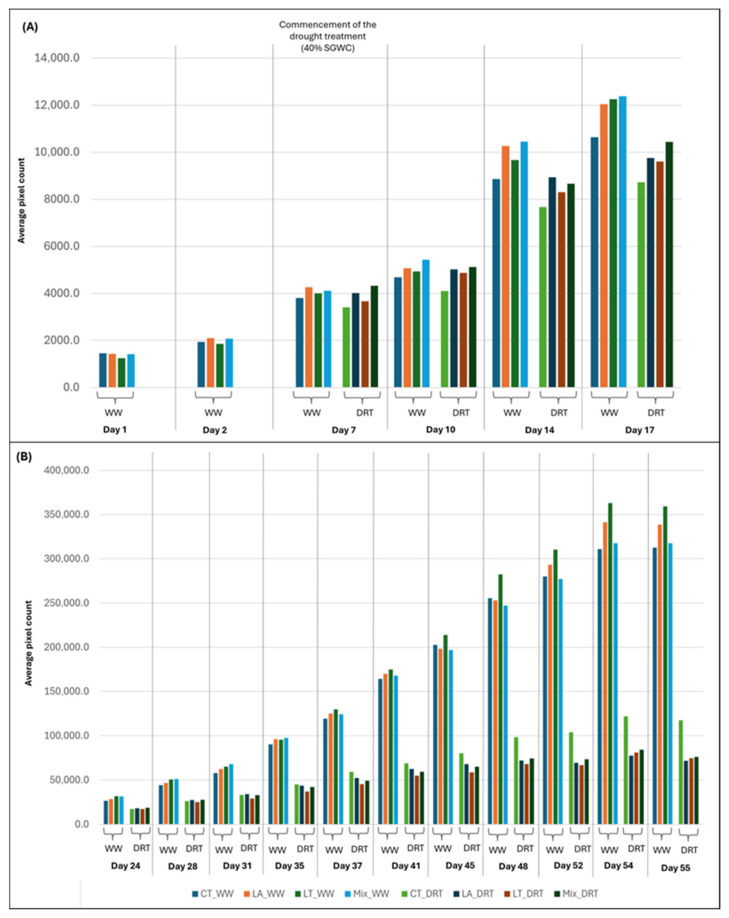
Temporal dynamics of aerial biomass accumulation in lucerne under well-watered and drought conditions. Bar plots show mean pixel-derived biomass values from HTP imaging. The treatments include uninoculated Control (CT), Laciniata (LA), Littoralis (LT), and Mix, each grown under well-watered (WW; 80% gravimetric water content [SGWC]) or drought (DRT; 40% SGWC) conditions. Drought stress was initiated seven days after the start of imaging at the PPV facility. (**A**) Early-phase growth responses (Day 1 to Day 17) prior to or during early drought onset. (**B**) Later-phase responses (Day 24 to Day 55), capturing the effects of prolonged watering regimes on biomass accumulation. Each bar represents the mean value per time point (*n* = 10 per treatment per watering condition).

To facilitate interpretation, the imaging data were divided into two temporal phases. The early phase comprised six imaging time points, during which all plants were uniformly irrigated. At Day 1 in the PPV, the Control (*n* = 20, mean = 1452.1) group exhibited the highest mean aerial biomass (digital volume), followed by LA (1428.9), Mix (1408.5), and LT (1245.1), indicating similar baseline growth across all treatments (Figure 9A). However, by Day 2, LA (mean = 2104.9, *p* = 0.157) and Mix (mean = 2074.4, *p* = 0.516)) treatments exceeded the Control (mean = 1936.5). Overall, LA treatment showed a trend toward enhanced growth. From Day 7 onwards, following the onset of differential watering, SynCom-specific effects became more pronounced. From Day 7 to Day 17 all SynCom treatments consistently outperformed the Control under both watering conditions, with the Mix treatment exhibiting the highest mean aerial biomass accumulation across this period. Time-series data for all early-phase time points are summarised in Supplementary Table S10. Day 17 at the PPV corresponds to 24 DAP in the conventional phenotyping timeline. Trends observed in HTP imaging values at this timepoint were consistent with destructive phenotyping results, where the Mix treatment also showed significantly greater shoot and root growth compared to the Control (Figure 6B; Supplementary Table S7). A significant drought effect was confirmed by Day 17 (*p* = 0.033), validating the effectiveness of the stress protocol and justifying continued analysis into the late phase.

#### 3.3.3. Late-Phase Imaging Analysis (Day 24 to Day 55)

The late phase comprised 11 imaging time points, during which half of the plants continued under well-watered conditions, and the remaining half were subjected to drought according to the defined drought protocol. Under well-watered conditions, all SynCom treatments exhibited higher biomass accumulation than the Control (n = 10), with the exception of three specific days. At Day 45 and Day 48, only the LT treatment exceeded the Control, while at Day 52, both LT and LA showed greater biomass than the Day 52 Control (Figure 9B; Supplementary Table S10).

Under drought conditions, all SynCom treatments outperformed the Control at Day 24. By Day 28, only the LA and Mix maintained higher aerial biomass than the Control, and by Day 31 this trend persisted exclusively in the LA treatment. From Day 35 onwards through to Day 55, the Control treatment exhibited the highest biomass across all treatments.

## 4. Discussion

### 4.1. A Holistic/Multifaceted Approach to Evaluating SynCom Efficacy on Lucerne Growth

This study employed an integrative phenotyping–microbiome framework to assess effects of SynComs, derived from *Medicago* wild relatives, on lucerne growth across multiple developmental stages under varying environmental and watering conditions. This framework was designed not only to capture plant growth responses but also to evaluate whether seed-derived SynComs from *Medicago* CWRs could modulate lucerne performance and microbiome assembly in an ecologically informed manner. Conventional phenotyping enabled precise, trait-specific measurements of root and shoot development, biomass accumulation, and recovery dynamics. In parallel, HTP imaging offered a non-destructive, time-resolved platform to monitor temporal growth trajectories with temporal resolution [41]. The combined use of these methodologies provided a comprehensive view of SynCom efficacy, capturing both early physiological responses and mid- to late-stage plant developmental outcomes. Similar dual-phenotyping strategies have been successfully applied in other crops, such as maize (*Zea mays*) [42], cotton [12], and soybean (*Glycine max*) [43]. The application of HTP in microbial inoculation studies has been valuable in sorghum [14,44] and maize [45], allowing precise quantification of temporal growth trends and plant–microbe interactions under different environmental conditions. In this context, the integration of microbiome profiling at 24 DAP further extended the analytical resolution of the study by linking early growth responses with microbial community dynamics. Collectively, this multifaceted approach enabled the evaluation of stage-specific SynCom performance, the functional relevance of microbial diversity within consortia, and treatment responses under both well-watered and drought conditions.

### 4.2. Functional Microbiome Restructuring Underpins Early Growth Responses

SynCom inoculation notably influenced early lucerne growth, with the Mix treatment yielding the highest germination rates, and significantly enhanced root and shoot elongation. These findings align with previous studies showing that microbial consortia can promote germination by breaking dormancy and accelerating metabolic activity, often through the production of phytohormones such as auxins (indole-3-acetic acid, IAA) and gibberellins (GA), which regulate root elongation and nutrient uptake [46,47,48,49]. Similar trends have been observed in wheat [50] and cotton [12], where SynCom application improved early growth through plant hormonal signalling and nutrient solubilisation. At 24 DAP, conventional phenotyping confirmed statistically significant improvements in root and shoot lengths under the Mix treatment, while HTP captured sustained aerial biomass gains from as early as 7 DAP (Figure 6B and Figure 9A). Together, these results suggest that early-stage plant responses are strongly influenced by microbial colonisation dynamics.

Microbiome analysis at 24 DAP revealed that SynCom inoculation altered microbial community composition in a treatment-specific way, reinforcing the hypothesis that early plant development is influenced by microbiome restructuring. Although alpha diversity indices did not show statistically significant differences, treatment-specific trends in microbial richness and evenness were evident (Figure 3). Notably, the Mix treatment, despite its superior growth effects, exhibited the lowest microbial richness among SynComs. These findings indicate that functional selectivity, rather than taxonomic diversity, is a key driver of early plant performance, likely shaped by competitive exclusion of non-beneficial microbes and priority effects, whereby early colonising taxa influence the assembly and function of the subsequent microbial community [51,52,53].

Indicator taxa analysis provides insights into microbial shifts associated with specific treatments, highlighting taxa that are selectively enriched or diminished following inoculation. These taxa are not the inoculated strains themselves, but rather native or coalesced microbes that respond predictably to treatment. As ecological markers, they reflect microbial adaptation, functional contributions to plant–microbe interactions, and potential mechanisms underlying SynCom-mediated plant benefits [12,54]. Identifying such taxa enables the evaluation of SynCom efficacy beyond overall microbial diversity, helping to pinpoint microbial groups involved in nutrient cycling, pathogen suppression, or microbiome stabilisation. The Mix treatment exhibited the most pronounced microbial shift, with Rhizobiales identified as the most dominant indicator group (Figure 5). This group is well known for biological nitrogen fixation, root colonisation, and phytohormone production, all contributing to enhanced plant growth. This dominance aligns with previous studies where SynCom inoculation enhanced plant growth through microbially driven nitrogen availability and metabolic interactions in the rhizosphere [55,56]. The dominance of Rhizobiales may have been shaped by a dynamic interaction between host selection and SynCom-mediated community shifts. Legume hosts are well known to preferentially enrich Rhizobiales due to their roles in nitrogen fixation and symbiosis [57,58], yet their consistent enrichment across SynCom treatments suggests that inoculation may also have facilitated ecological conditions favouring their persistence. Thus, both plant-driven filtering and SynCom influence may have contributed to the observed outcome. In addition, the Mix treatment showed enrichment of Streptomycetales, while Vermiphilaceae and Kapabacteriales, which were detected in the other SynCom treatments and the Control, were notably absent. These patterns suggest that the Mix SynCom exerted distinct selective pressures, leading to a more streamlined yet functionally optimised microbiome.

Core microbiome analysis provides further evidence that treatment-specific microbial assemblages influence plant responses. Across all treatments, 137 ASVs were consistently shared (Figure 4A), suggesting that a stable core microbiome persists regardless of SynCom inoculation. These taxa likely represent ubiquitous microbial members of the plant microbiome with conserved ecological roles. Key shared taxa included *Pseudomonas*, *Bradyrhizobium*, ANPR-complex, *Massilia*, *Novosphingobium*, and *Streptomyces*, which are broadly associated with nitrogen fixation, plant growth promotion, and stress resilience [59,60,61,62,63]. Their consistent presence across treatments suggests that SynComs integrate with native microbiomes rather than fully displacing them, contributing to microbial coalescence and influencing community assembly through priority effects [52]. Beyond the shared core microbiome, a total of 149 ASVs were detected exclusively in SynCom-treated plants but were absent from the Control, indicating that SynCom inoculation selectively recruited or maintained certain microbes. Notable taxa among these included *Azospirillum*, *Sphingomonas*, Burkholderiales, *Flavobacterium*, and *Streptomyces*, which contribute to nutrient cycling, pathogen suppression, and microbiome stability [61,64,65,66,67]. These findings align with previous studies demonstrating that SynComs facilitate beneficial microbial interactions that improve plant establishment [12,68]. These compositional trends also align with alpha diversity patterns. LT-treated plants exhibited both higher species richness and a greater number of unique core ASVs, suggesting broad microbial recruitment or retention. In contrast, the Mix SynCom (combination of LA and LT consortia), despite its superior growth effects, supported a more compositionally constrained microbiome, with fewer unique ASVs and lower diversity indices. This pattern may reflect a process of functional streamlining, whereby a narrower set of taxa maintain community performance by fulfilling essential roles. Such dynamics are consistent with keystone taxa theory, which proposes that certain microbes exert disproportionate functional influence relative to their abundance [69]. In the context of SynCom design, this suggests that ecological coherence and the retention of functionally pivotal taxa may be more critical than maximising taxonomic richness for achieving reliable plant benefits.

Distinct patterns in microbiome composition were observed across SynCom treatments. The LA-inoculated plants were enriched in *Novosphingobium* [70] and *Thiomonas* [71], which have been associated with nutrient cycling and soil health, albeit with less well-defined roles in plant growth. In contrast, the LT treatment exhibited the highest microbial richness, yet this was not reflected in enhanced plant growth. This divergence further supports the interpretation that taxonomic diversity alone does not equate to functional efficacy. Beyond general shifts in microbial diversity and core community composition, attention was also given to taxa uniquely associated with the three SynCom treatment groups, as these may provide insights into functional contributions to growth and stress resilience. A total of 12 ASVs—including *Bdellovibrio*, *Pedosphaera*, and unclassified Rhizobiaceae—were uniquely detected in SynCom-treated plants and were absent from the Control. *Bdellovibrio* is a known bacterial predator with potential roles in pathogen suppression [72], while *Pedosphaera* contributes to soil health and organic matter decomposition [73]. Such functions may indirectly enhance lucerne growth by reducing pathogen pressure, improving nutrient turnover, and stabilising the rhizosphere community under stress.

While these findings demonstrate clear microbiome compositional shifts at 24 DAP, it remains unclear whether such restructuring persisted into later stages or contributed directly to plant performance. Sequencing was not extended beyond this point, but the drought-related trends observed at later stages suggest that early inoculation may have left a lasting influence, since SynCom treatment was the only factor separating plants. These aspects are discussed in the following sections.

### 4.3. Temporal Dynamics of SynCom-Mediated Growth Under Contrasting Water Regimes

At 63 DAP, an intermediate growth stage of lucerne, biomass accumulation was assessed under well-watered and drought conditions to evaluate SynCom-mediated plant responses prior to maturity. Conventional phenotyping revealed no statistically significant differences among treatments; however, SynCom-inoculated plants generally exhibited higher biomass than the Control, particularly under well-watered conditions (Figure 7). The lack of statistical significance at this stage likely reflects biological variability and modest effect sizes, consistent with trends observed across treatments. HTP imaging further supported this trend, capturing temporal growth dynamics not fully resolved by conventional phenotyping. Under well-watered conditions, all SynCom treatments outperformed the Control at specific time points, with LT exhibiting the most consistent performance (Figure 9). This may be attributed to the enrichment of Cytophagales, Chitinophagales, and Saccharimonadales observed at 24 DAP, taxa known to enhance nutrient availability, organic matter accumulation, microbiome stability, and hormone regulation, potentially contributing to sustained biomass production [74,75,76]. These findings align with previous studies demonstrating that microbial consortia can improve biomass accumulation by facilitating nutrient uptake, modulating hormone signalling, and optimising root system architecture under favourable conditions [43,50,77,78].

Under drought conditions, SynCom treatments did not confer a statistically significant biomass advantage over the Control. Interestingly, Control plants exhibited higher biomass at later time points, which may be partially explained by the elevated relative abundance of *Azospirillum* in the Control microbiome at 24 DAP. *Azospirillum* spp. have been widely reported to support drought adaptation through mechanisms such as osmoprotectant production (e.g., proline and betaine), modulation of root architecture, and regulation of phytohormones (auxins, GA, and cytokinins) [79,80]. These functions, documented in other crops, are thought to contribute to improve water-use efficiency and stress mitigation, and may provide a partial explanation for the performance of Control plants at later stages. Additionally, *Azospirillum* and related taxa are known for nitrogen fixation, phosphorus solubilisation, and ACC deaminase activity, as well as biofilm formation that stabilises their rhizosphere presence [79]. Such traits have been linked with improved stress resilience in cereals such as maize and wheat [79,81], and may be relevant to lucerne, although they were not directly tested in the current study.

Among SynCom-treated plants, the LA treatment sustained higher biomass accumulation for up to 24 days after drought initiation, supporting the hypothesis that drought-adapted microbes from *M. laciniata* may contribute to early stress resilience. Microbiome profiling at 24 DAP aligns with this interpretation, as LA-treated plants exhibited the highest relative abundance of *Azospirillum* among SynComs, along with enrichment in Sphingomonadales and Burkholderiales—microbial groups previously associated with drought adaptation in crops such as wheat and sugarcane (*Saccharum officinarum*) [80,82,83]. These taxa have been linked with improved water-use efficiency, regulation of stress-responsive signalling, and beneficial root–microbe interactions in other systems, and may have contributed to the modest biomass retention observed in LA-inoculated plants under drought, although this requires direct functional validation. Beyond Day 35, Control plants maintained the highest aerial biomass, suggesting that intrinsic plant adaptation mechanisms or possible unintended competitive interactions between SynCom members and native drought-adapted microbes may underlie long-term drought resilience. As lucerne is primarily grown as a pasture legume, aerial biomass provides the most relevant indicator of agronomic performance. The transient performance advantage observed in LA-treated plants under drought highlights an important consideration: early SynCom benefits may not always translate into prolonged functional gains under persistent abiotic stress. This underscores the need to assess SynCom performance across developmental stages and stress durations to capture their full potential.

More broadly, these observations reinforce the complexity of plant–microbiome–environment interactions. SynCom performance is shaped not only by inoculant composition, but also by compatibility with host genotype and environmental context—a relationship conceptualised as G_P_ × G_M_ × E [1,2,3], as outlined in the introduction. Future optimisation of SynComs will require designs that incorporate this dynamic triad, particularly under variable field conditions where microbial contributions to stress resilience are likely to shift over time.

Exploratory phenotyping at 76 DAP (*n* = 3 per treatment) was also conducted to assess post-stress recovery. Due to the limited replication, these data were not used for statistical comparisons but provided descriptive insights into treatment-specific recovery dynamics. Full details are presented in Supplementary Section S7.

### 4.4. Insights from Phenotyping and Microbiome Approaches in Evaluating SynCom Efficacy

In addition to the biological interpretations discussed above, this study offers reflections on the methodological framework used to evaluate SynCom efficacy. Although microbiome analysis was conducted at two time points (24 and 76 DAP), only the 24 DAP data were included in the final analysis due to limited replication at 76 DAP (*n* = 3). Future studies would benefit from increased replication to improve the reliability of microbiome inferences and to better capture temporal shifts. Nevertheless, integrating early microbiome data with time-resolved phenotyping enabled a multi-dimensional interpretation of SynCom effects across developmental stages and watering regimes, offering a scalable and informative framework for evaluating SynCom efficacy in diverse plant systems.

The integration of conventional and HTP methods enabled a comprehensive assessment of SynCom performance in lucerne. Conventional phenotyping facilitated precise, trait-specific measurements at key developmental stages, offering valuable insights into root and shoot responses to inoculation. However, its labour-intensive nature and limited scalability constrained the ability to detect fine-scale growth dynamics and treatment responses over time [41,84]. By contrast, HTP imaging allowed for non-destructive, high-resolution biomass estimation, enabling the detection of temporal trends and subtle treatment-induced effects that conventional phenotyping could not fully resolve [85,86]. Imaging sensor-based HTP platforms have demonstrated robust performance across diverse crops such as rice [87], maize [33], and wheat [88], and the strong correlation observed in the study between pixel-derived biomass and conventional wet biomass (Figure 8) affirms the reliability of this approach [41]. The ability of HTP to resolve stage-specific SynCom effects, particularly between 7 DAP to 63 DAP, underscores its utility in microbial efficacy studies, where automation and time-resolved measurements are critical [45,84,86]. Capturing these temporal responses enabled a deeper understanding of SynCom performance under both optimal and stress conditions [89]. While HTP imaging proved valuable for monitoring shoot development, several limitations warrant consideration. Root traits were not captured, and pixel-based biomass estimates can be influenced by variability in plant architecture or image noise. However, potential confounding effects due to uneven lighting conditions were minimised through the randomised layout of plants in the PPV glasshouse; replicates from each treatment were spatially distributed rather than grouped together, thereby reducing potential bias. Nevertheless, the clear and reproducible trends observed suggest that HTP platforms remain highly useful when complemented with conventional measurements and well-defined experimental controls.

## 5. Conclusions

This study advances a new paradigm for SynCom design by prioritising ecological relevance over trait-based assembly at the proof-of-concept stage. By constructing SynComs exclusively from seed-associated microbes of *Medicago* CWRs and selecting strains with high genomic similarity to core taxa in lucerne, we demonstrate that evolutionarily aligned, ecologically co-occurring microbial consortia can modulate early plant growth and restructure the host microbiome assembly. Rather than acting solely as biofertilisers, these SynComs appeared to function as ecological filters, selectively shaping microbial communities in ways that were consistent with early-stage phenotypic responses. Importantly, our findings challenge the assumption that greater microbial diversity inherently leads to better plant outcomes [90]. Instead, we provide early evidence that SynComs with lower taxonomic richness but stronger ecological coherence may deliver more consistent effects. These results reposition SynComs not as additive tools, but as targeted interventions that engage selectively with host microbiomes during critical developmental windows. The integration of conventional phenotyping, HTP-imaging, and early microbiome profiling enabled a time-resolved view of SynCom efficacy. While no statistically significant improvements were observed under drought conditions, biological trends, together with treatment-specific microbiome restructuring, support the value of evaluating SynCom performance through ecological and community-level metrics—not just biomass. Although the study was limited by robust sequencing at a single early time point and modest replication at later harvests, the reproducibility of SynCom-driven trends across experiments supports the utility of this experimental framework.

Future work should extend longitudinal microbiome profiling to track SynCom persistence, apply strain-resolved methods such as metagenomics, qPCR tagging, or transcriptomics to identify mechanistic drivers of host responses, and integrate direct physiological measurements of plant stress and nutrition. Expanding trials across diverse *Medicago* genotypes and field environments will be essential to evaluate scalability and robustness. Moreover, refining seed-coating and delivery platforms will support translational deployment under real world conditions.

More broadly, this work lays a conceptual and methodological foundation for next-generation SynCom design—one that leverages ecological co-occurrence, evolutionary compatibility, and host–microbiome continuity to guide targeted microbiome interventions in agriculture. It highlights the untapped potential of CWRs not only as genetic resources but also as microbial reservoirs that can inform the development of seed-based solutions for sustainable crop production. In the face of climate variability, declining soil health, and rising input costs, microbiome-informed strategies such as these offer a timely and resilient path forward, supporting stress resilience and productivity in forage systems and beyond. By linking ecological insight to SynCom design and evaluation, this study provides early evidence to guide the development of microbial tools for more sustainable agriculture.

## Figures and Tables

**Table 1:** Taxonomic identity and genomic features of seed-derived bacterial isolates used in SynCom design.

Bacterial Isolate Lab ID and NCBI Accession Number	Isolation Source (Host Plant Species)	Taxonomic Classification (Kraken Database)	ANI %	NCBI Reference Genome ID
Lu_LA164_003 (PQ756890)	*M. laciniata*	*Pantoea agglomerans* pv. *betae*	97.41%	*Pantoea agglomerans* (GCF_019048385.1)
Lu_LA841_009 (PQ756893)		*Pantoea allii*	99.80%	*Pantoea allii (GCF_003148935.1)*
Lu_LA164_012 (PQ756889)		*Pseudomonas graminis*	85.93%	*Pseudomonas graminis* DSM 11,363 (GCF_900111735.1)
Lu_LT198_003 (PQ756897)	*M. littoralis*	*Pantoea agglomerans* pv. *betae*	97.43%	*Pantoea agglomerans* (GCF_019048385.1)
Lu_LT198_002 (PQ756896)		*Pantoea allii*	80.43%	*Pantoea allii* (GCF_003148935.1)
Lu_LT198_W003 (PQ756901)		*Pseudomonas graminis*	99.85%	*Pseudomonas graminis* DSM 11,363 (GCF_900111735.1)

Isolates were taxonomically classified using Kraken2 with the NCBI RefSeq bacterial genome database (build date: March 2023). Average Nucleotide Identity (ANI) was calculated against the closest NCBI reference genome. The NCBI reference genome ID and the GenBank accession numbers of each isolate are provided for reproducibility.

**Table 2:** Core microbiome composition and statistical comparison of ASVs overlap across treatments. Statistical significance *p* < 0.001.

Control vs. SynCom-Treated Groups					
Comparison	Shared Taxa	Unique to Control	Unique to Treatment	Fisher’s Exact Test	Chi-Square Test
CT vs. LA	72.60%	11.10%	16.30%	0.1958	0.2331
CT vs. LT	65.70%	3.60%	30.70%	3.02 × 10^−12^	8.18 × 10^−11^
CT vs. Mix	66.80%	12.30%	20.90%	0.0502	0.0626
**Among SynCom-Treated Groups**					
LT vs. LA	67.45%	27.50% (LT)	5.10% (LA)	6.14 × 10^−9^	2.97 × 10^−8^
LT vs. MIX	65.40%	26.60% (LT)	8.00% (Mix)	3.09 × 10^−6^	7.52 × 10^−6^
LA vs. MIX	68.00%	14.22% (LA)	17.78% (Mix)	0.4331	0.4731

## Data Availability

The original data presented in the study is openly available in the NCBI GenBank under the BioProject PRJNA1180717.

## Supplementary Materials

The following supporting information can be downloaded at https://www.mdpi.com/article/10.3390/microorganisms13092114/s1. Table S1: Constituent bacterial isolates of the synthetic communities (SynComs) designed in the study; Table S2: Summary of 16S rRNA gene sequencing and rarefaction results for samples collected at 24 days after planting (DAP) in the glasshouse (Bundoora, Victoria, Australia); Table S3: Amplicon sequence variants (ASVs) shared across all SynCom treatments and Control groups based on 16S rRNA gene profiling at 24 days after planting (DAP); Table S4: Amplicon sequence variants (ASVs) shared across all SynCom-treated groups based on 16S rRNA gene profiling at 24 days after planting (DAP); Table S5: Amplicon sequence variants (ASVs) uniquely detected in SynCom-treated groups based on 16S rRNA gene profiling at 24 days after planting (DAP); Table S6: Order-level indicator taxa and their relative abundance (%) in SynCom-treated and Control plants at 24 DAP; Table S7: Descriptive statistics summary of root and shoot measurements across treatment groups at 24 DAP; Table S8: Descriptive statistics summary of aerial biomass at 63 DAP across treatment groups and watering conditions; Table S9: Descriptive statistics (means ± SD) of growth parameters at 76 DAP under post-stress recovery conditions for different treatment groups and watering conditions; Table S10: Mean adjusted pixel counts (digital volume) across treatments under both well-watered (WW) and drought (DRT) conditions at 17 time points, measured using the LemnaTec 3D Scanalyser at the Plant Phenomics Victoria (PPV) facility. Figure S1: Validation of image-derived biomass estimates against conventional biomass measurements at 63 DAP. Bar plot showing individual-level comparisons of adjusted pixel values and wet biomass for lucerne plants under different SynCom treatments and watering regimes. Each bar represents a single biological replicate (*n* = 5 per treatment), with values grouped by treatment and condition (well-watered and drought). Adjusted pixel values were derived from LemnaTec Scanalyser 3D imaging, and wet biomass was recorded via destructive phenotyping. Data were collected at the PPV facility; Figure S2: Root and shoot growth responses of lucerne seedlings at 76 DAP under different SynCom treatments. (A) Root length, (B) root biomass, (C) shoot length, and (D) shoot biomass were measured in plants treated with Control and SynCom treatments: Laciniata, Littoralis, and Mix. Bars represent mean ± standard error (SE). Measurements indicate post-stress recovery dynamics following different SynCom inoculations; Figure S3: Fold change analysis of plant growth across developmental stages. (A) Root and shoot length fold changes from 24 DAP and 76 DAP across Control, Laciniata, Littoralis, and Mix treatments. Fold changes were calculated as the ration of mean measurements at 76 DAP relative to 24 DAP. (B) Fold changes in wet aerial biomass between 63 DAP to 76 DAP across treatments. Bars represent mean fold change values; Section S1: The composition of the standard potting mix used in glasshouse and plant phenomics studies; Section S2: Soil gravimetric water content (SGWC) determination and drought application; Section S3: HTP image analysis; Section S4: DNA extraction, 16S rRNA amplicon library preparation and sequencing; Section S5: Evaluating the effects of SynCom treatments on plant growth and biomass at 76 DAP under post-stress recovery conditions; Section S6: Comparison of growth parameters across time-points; Section S7: SynCom performance during post-stress recovery and later growth stages. Tables S3–S5 are provided as separate excel files and are accessible via the Zenodo repository (https://doi.org/10.5281/zenodo.16355480). Additional references cited exclusively in the Supplementary Materials are included in the reference list for completeness [91,92,93,94,95,96,97,98].

## Abbreviations

The following abbreviations are used in this manuscript:

16S rRNA	16S ribosomal ribonucleic acid
ANI	Average nucleotide identity
ANOVA	Analysis of variance
ANPR	*Allorhizobium–Neorhizobium–Pararhizobium–Rhizobium*
ASV	Amplicon sequence variant
CWRs	Crop wild relatives
DAP	Days after planting
DNA	Deoxyribonucleic acid
GA	Gibberellins
HTP	High-throughput phenotyping
IAA	Indole-3-acetic acid
LA	Laciniata SynCom (three-strain SynCom from *M. laciniata*)
LT	Littoralis SynCom (three-strain SynCom from *M. littoralis*)
Mix	Mix SynCom (six-strain SynCom combining LA and LT)
NB	Nutrient broth
NCBI	National Centre for Biotechnology Information
PPV	Plant Phenomics Victoria
QIIME2	Quantitative Insights Into Microbial Ecology 2
SGWC	Soil gravimetric water content
SynComs	Synthetic communities
