# Supplementary material for: Seed-Derived Synthetic Microbial Communities (SynComs) from Medicago Wild Relatives Modulate Early Plant Microbiome Assembly and Phenotypic Traits in Lucerne (Medicago sativa L.)
Source: Microorganisms. 2025 Sep 10;13(9):2114. doi: 10.3390/microorganisms13092114 (PMC12472412; doi:10.3390/microorganisms13092114)
Supplement: Supplementary file 1 [file microorganisms-13-02114-s001.zip › microorganisms-3805004-supplementary.pdf]

Seed-derived synthetic microbial communities (SynComs) from *Medicago* wild relatives modulate early plant microbiome assembly and phenotypic traits in lucerne (*Medicago sativa* L.)

Supplementary Materials

Supplementary Table S1. Constituent bacterial isolates of the synthetic communities (SynComs) designed in the study

SynCom pool name	Laciniata (LA) pool	Littoralis (LT) pool	Mix pool
SynCom isolates	Lu_LA164_003<br>Lu_LA841_009<br>Lu_LA164_012	Lu_LT198_003<br>Lu_LT198_002<br>Lu_LT198_W003	Lu_LA164_003<br>Lu_LA841_009<br>Lu_LA164_012<br>Lu_LT198_003<br>Lu_LT198_002<br>Lu_LT198_W003

Supplementary Table S2. Summary of 16S rRNA gene sequencing and rarefaction results for samples collected at 24 days after planting (DAP) in the glasshouse (Bundoora, Victoria, Australia)

Metric	Value
Total raw sequencing reads	43,159,116
Reads retained after quality filtering	10,461,259
Reads retained after chloroplast and mitochondrial sequence removal	9,521,338
Total samples prior to rarefaction	80
Rarefaction depth applied	20,000 reads/sample
Total reads retained post-rarefaction	940,000
Number of samples retained post-rarefaction	47
Total amplicon sequence variants (ASVs) retained post-rarefaction	3,299
Minimum read count before rarefaction	0
Maximum read count before rarefaction	504,125
Median read count before rarefaction	28,893

Supplementary Table S6. Order-level indicator taxa and their relative abundance (%) in SynCom-treated and Control plants at 24 DAP

Order	Control	Laciniata	Littoralis	Mix
Azospirillales	79.33	38.14	19.01	10.62
Cytophagales	0.39	1.71	9.20	1.49
Chitinophagales	2.58	3.59	8.82	0.71
SBR1031	2.35	3.59	7.93	1.65
Saccharimonadales	0.95	0.98	7.05	0.12
Legionellales	0.45	0.73	6.94	0.08
Rhizobiales	0.84	2.12	6.39	71.01
Sphingomonadales	7.61	36.19	5.01	3.66
Propionibacteriales	0.67	1.30	4.79	0.51
Burkholderiales	0.22	4.56	3.64	0.39
Gemmatimonadales	0.56	0.81	2.81	0.20
Chthoniobacterales	0.67	0.33	2.48	0.08
Micrococcales	0.62	0.24	2.09	0.67
Pedosphaerales	0.06	0.00	2.09	0.08
CCD24	0.90	0.57	1.93	0.16
Defluviicoccales	0.00	0.00	1.76	0.00
Pirellulales	0.00	0.00	1.49	0.00
Vicinamibacterales	0.45	0.24	1.43	0.16
Babeliales	0.62	1.55	1.43	0.00
Gammaproteobacteria_Incertae_Sedis_Unknown_Family_Acidibacter	0.06	0.33	0.99	0.04
Polyangiales	0.00	0.08	0.88	0.00
Streptomycetales	1.12	0.65	0.77	7.51
Kapabacteriales	0.06	0.24	0.76	0.00
Xanthomonadales	0.17	2.04	0.17	0.12
Oligoflexales	0.00	0.00	0.17	0.00
Thermomicrobiales	0.00	0.00	0.00	0.47
Frankiales	0.00	0.00	0.00	0.28

Supplementary Table S7. Descriptive statistics summary of root and shoot measurements across treatment groups at 24 DAP

Treatment	Tissue	Mean (mm)	SD	SE
Control	Root	95.00	20.43	6.46
Laciniata		100.80	20.14	6.37
Littoralis		86.20	11.59	3.67
Mix		123.70	27.70	8.76
Control	Shoot	143.50	37.30	11.80
Laciniata		167.80	31.15	9.85
Littoralis		154.30	24.82	7.85
Mix		186.10	28.98	9.16

Supplementary Table S8. Descriptive statistics summary of aerial biomass at 63 DAP across treatment groups and watering conditions

Treatment	Watering condition	Wet weight (g)			Dry weight (g)		
		**Mean**	**SD**	**SE**	**Mean**	**SD**	**SE**
CT	Well_watered	10.94	6.22	2.78	1.96	1.29	0.58
LA		12.36	2.23	1.00	2.13	0.30	0.13
LT		13.71	4.82	2.16	2.33	0.95	0.42
Mix		12.46	4.62	2.07	2.28	1.08	0.48
CT	Drought	3.78	1.56	0.70	0.80	0.33	0.15
LA		2.86	1.64	0.74	0.53	0.26	0.12
LT		2.95	1.66	0.74	0.66	0.24	0.11
Mix		2.83	0.87	0.39	0.59	0.17	0.08

Supplementary Table S9. Descriptive statistics (means ± SD) of growth parameters at 76 DAP under post-stress recovery conditions for different treatment groups and watering conditions.

Treatment group	Well-watered watering condition							
	**RL (cm)**		**RW (g)**		**SL (cm)**		**SW (g)**	
	Mean	SD	Mean	SD	Mean	SD	Mean	SD
Control	31.43	2.21	19.76	4.65	50.53	8.55	21.88	1.92
Laciniata	36.13	2.15	25.00	4.42	51.07	11.06	26.06	2.63
Littoralis	31.57	4.76	33.25	15.17	47.27	2.36	22.24	2.10
Mix	34.70	3.03	24.52	6.62	45.63	2.01	24.35	4.97
	**Drought watering condition**							
Control	30.40	1.98	7.47	2.33	30.80	4.38	9.85	4.83
Laciniata	33.73	0.97	4.57	4.25	28.87	10.91	5.84	2.63
Littoralis	32.57	4.30	10.39	11.03	24.77	8.72	9.54	3.70
Mix	35.77	2.27	5.09	2.76	24.67	5.47	6.58	2.86

*RL, root length; RW, root weight; SL, shoot length; SW, shoot weight

Supplementary Table S10. Mean adjusted pixel counts (digital volume) across treatments under both well-watered (WW) and drought (DRT) conditions at 17 time points, measured using the LemnaTec 3D Scanalyser at the Plant Phenomics Victoria (PPV) facility.

**Time point at PPV**	**CT_WW**	**LA_WW**	**LT_WW**	**Mix_WW**	**CT_DRT**	**LA_DRT**	**LT_DRT**	**Mix_DRT**
Day 1	1484.00	1477.70	1214.80	1456.00	1420.20	1380.00	1275.30	1380.90
Day 2	1854.78	2153.70	1855.50	2034.70	2018.20	2056.00	1855.10	2114.00
Day 7	3802.00	4261.60	3997.50	4114.30	3403.00	4014.20	3670.00	4327.20
Day 10	4689.78	5074.00	4934.40	5428.20	4095.30	5018.60	4876.00	5120.20
Day 14	8859.00	10263.40	9669.20	10446.10	7660.40	8930.70	8298.40	8665.80
Day 17	10636.33	12034.20	12255.50	12377.10	8727.00	9755.80	9603.50	10434.10
Day 24	26646.00	28669.60	31845.40	31580.70	17271.20	18158.70	17385.40	18850.00
Day 28	44301.11	46965.30	50552.10	51162.70	26198.90	27475.90	25232.50	27827.60
Day 31	57996.22	62656.50	64995.90	67926.70	33314.20	34198.30	29275.50	33120.90
Day 35	90367.56	96109.70	95545.10	97416.70	45133.30	43783.90	37077.90	42232.50
Day 37	119215.33	125184.40	129714.70	124081.10	59306.80	52483.60	45359.40	49207.60
Day 41	164090.89	169940.70	174746.10	167935.50	69018.10	62408.50	55037.60	59198.60
Day 45	202566.78	198330.70	213988.70	196825.10	80065.80	68133.30	58763.70	65013.60
Day 48	255531.78	252997.50	282509.60	247169.00	98404.70	72151.50	68065.40	74501.50
Day 52	280402.33	293193.80	310311.90	277505.10	104064.20	69339.40	67002.10	73589.10
Day 54	311059.33	341322.80	363227.40	317609.30	122005.40	77653.60	81089.70	84382.60
Day 55	312685.56	338703.70	359346.80	317500.40	117657.10	71911.70	74690.20	76005.80

OFFICIAL

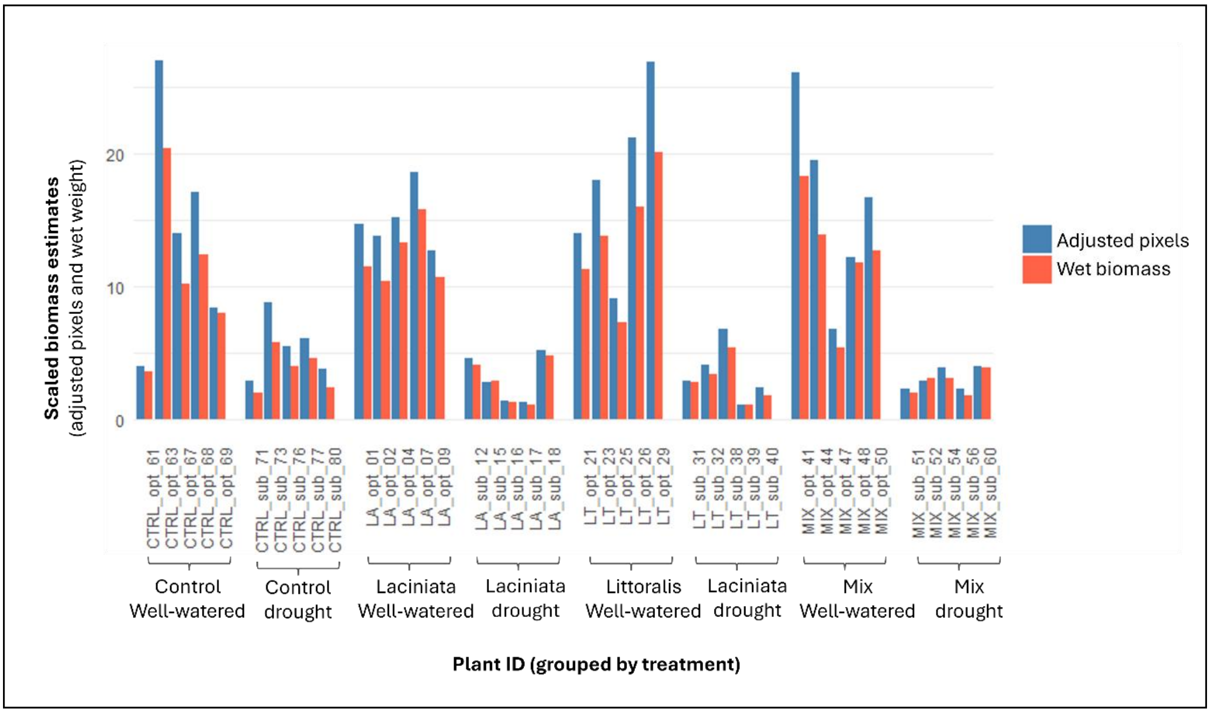

Supplementary Figure S1. Validation of image-derived biomass estimates against conventional biomass measurements at 63 DAP. Bar plot showing individual-level comparisons of adjusted pixel values and wet biomass for lucerne plants under different SynCom treatments and watering regimes. Each bar represents a single biological replicate (n = 5 per treatment), with values grouped by treatment and condition (well-watered and drought). Adjusted pixel values were derived from LemnaTec Scanalyser 3D imaging, and wet biomass was recorded via destructive phenotyping. Data were collected at the PPV facility.

Supplementary Section S1

The composition of the standard potting mix used in glasshouse and plant phenomics studies.

Base material	Target %	Target m^3
Medium-deep mined sand	20	8.8
Peat moss	5	2.2
CB0306*	35	15.4
NB0006*	45	19.8
Coco peat	5	2.2

*a proprietary composted organic material supplied by (Van Schaik's Bio Gro Pty. Ltd., Dandenong South, Victoria, Australia)

In addition to the base components, the mix was supplemented with trace elements and fertilisers at the following rates.

Trace elements	Kg/m^3
Fine lime-1393	2.5
Dolomitp7090	1.0
Gypsum-215	2.0
Hydralene-MU200	0.7
Calcium nitrate + boron	0.3
Micro-Mix 240	0.5
Nitrolene 39-mu001	0.3
Superphosphate (Fine-44)	0.5
Potassium sulphate-325	0.5
Ferrous sulphate-p7050	0.5

The potting mix was pH-adjusted to a target range of 5.30–6.50, with lab-measured pH of 5.56 and electrical conductivity (EC) of 1.11. Nutrient analysis showed nitrate levels at 45 mg/kg and ammonium below 20 mg/kg. Fertiliser and trace element blending was performed by Bio Gro staff and verified by in-house quality control.

Supplementary Section S2

Soil gravimetric water content (SGWC) determination and drought application

SGWC determination and target mass calculation

Soil gravimetric water content (SGWC) was used to standardise watering across treatments and ensure consistent drought application. SGWC represents the amount of water in the potting mix as a percentage of its total water-holding capacity. In this study, 80% SGWC was selected for well-watered conditions, while 40% SGWC was used for drought treatments. The target mass required to achieve a specific SGWC was calculated using the equation:

$$m_{target} = \left(\frac{m(saturated) - \left(\frac{\sum_{i=1}^{n} m(dry)_i}{n} \right)}{100} \times m_{(target\ \%)} \right) + \left(\frac{\sum_{i=1}^{n} m(dry)_i}{n} \right) + k$$

where,

- $m(saturated)$ = mass of the pot at full water-holding capacity (g)
- $\frac{\sum_{i=1}^{n} m(dry)_i}{n}$ = mean dry mass of the potting mix (g)
- $m_{(target\ \%)}$ = target SGWC as a percentage
- k = combined mass of the pot (102 g), saucer (41 g), cage (282 g), and carrier (2235

Since different potting mixes have varying air porosity and water-holding capacities, saturated and dry masses were determined empirically for the potting mix used in this study.

Determination of saturated and dry mass of potting mix

To calculate the saturated and dry mass, six to ten replicate pots were filled with the experimental potting mix and weighed to determine their empty pot mass. The pots were then:

- Saturated: water was applied until drainage ceased, and the mass of each pot was recorded after 30 minutes of draining.
- Dried: the potting mix was removed, oven-dried at 65 °C for one week, and weighed again to determine the dry mass.

These values were averaged to derive the mean saturated and dry mass, which were then used in SGWC calculations.

Automated watering and drought application

Watering was automated using the LemnaTec Scanalyser 3D system at PPV, Horsham. The system automatically weighed pots before each watering event, ensuring individualised application based on target SGWC. Well-watered plants were maintained at 80% SGWC, receiving water twice daily to compensate for evapotranspiration. Drought-treated plants were acclimated under 80% SGWC for seven days before watering was reduced to 40% SGWC once

daily for the remainder of the experiment. This approach ensured precise, reproducible drought application, minimising variability due to environmental fluctuations.

Systematic considerations for soil handling and experimental design

1. To prevent anoxia and maintain consistent soil structure: saucer trays were placed under each pot to prevent waterlogging and protect conveyor systems.
2. The potting mix was not compacted before planting to maintain uniform soil aeration.
3. The effect of soil slackening (settling over time) was tested by pre-watering additional pots over two weeks to measure any significant loss in soil height.

These methods ensured that all experimental plants experienced comparable soil moisture dynamics, supporting reliable phenotypic assessments.

Supplementary Section S3

HTP image analysis

Plants were imaged twice per week on the LemnaTec Scanalyser 3D automated phenomics platform via a series of visible-spectrum (red–green–blue, RGB) cameras (Prosilica GT, Allied Vision Technologies GmbH, Stadtroda, Germany) fitted with a 50 mm focal lens (T* 250 ZF, Carl Zeiss AG, Oberkochen, Germany), located within an imaging cabinet to control lighting conditions. RGB images of each plant were taken from above (top view, TV) and from the side at three angles (0°, 120°, 240°). Snapshot images were stored as blob files within a PostgreSQL database. A modified version of the PlantCV 'Data Science Tools' module was used to extract each snapshot as a series of six 24-bit Portable Network Graphic (PNG) images (4384 × 6576 pixels). A customised PlantCV [1] analysis pipeline was then developed to carry out the image analysis. This was run using PlantCV version 4.5.1, OpenCV version 4.10.0.84, and Python version 3.12.10 within a CentOS Linux version 7.9.2009 environment on the Biosciences Advanced Scientific Computer (BASC) cluster at the Centre for AgriBioscience in Bundoora, Victoria, Australia.

For each snapshot, PNG images were read into the pipeline and processed. The images were cropped to isolate plants from the side-view, to standardise them for further processing. Side-view RGB images were converted to CMYK colour space (cyan, magenta, yellow, and black bands), where the Y channel was particularly effective in isolating the plant from its background under constant lighting conditions across all images, by applying a fixed grey-level threshold of 71. The C channel was used to detect the blue plant support cage via a threshold of 75, followed by a 5 × 5 dilation, while the L channel enabled isolation of the carrier (potholder) using a threshold of 41 and the same dilation step. These binary masks (cage and carrier) were combined using a logical-OR operation. The carrier plate, located at the top of the carrier, was isolated using a threshold of 31 on the M channel, followed by a 5-pixel morphological opening, and added to the previous mask using another logical OR operation. This composite mask was used to subtract the carrier, cage and cage plate from the plant mask. Finally, a 3 × 3 dilation was applied to the binary mask to define the final region of interest for further analysis. To isolate plants from top-view images, RGB images were converted to l*a*b colour space. The *a channel allowed for clear plant–background separation using a fixed grey-level threshold of 120, followed by inversion. To exclude tracks, carrier and potting mix from the imaging chamber, the RGB images were also converted to HSV (hue, saturation,

value) colour space, and a threshold of 60 was applied. Two rounds of 9 × 9 dilations were then performed to generate a binary mask, which was subtracted from the top-view plant mask to accurately identify a foreground region-of-interest for top-view images [2].

Digital volume, calculated as the sum of green pixel areas from three side views and one top view per time point, was used as a proxy for biomass. This metric, derived using the $V_{LemnaTec}$ formula [3], has been independently validated to correlate with plant biomass.

Supplementary Section S4

DNA extraction, 16S rRNA amplicon library preparation and sequencing

Lucerne plants were harvested at two time points, 24 DAP (10 replicates per treatment) and 76 DAP (3 replicates per treatment) for 16S rRNA gene sequencing. Plants were carefully uprooted, and loosely adhered soil was removed. They were then dissected into root and shoot (aboveground tissue) sections. For DNA extraction, three to four tissue fragments (~0.5 cm^2 each) were collected from each section, placed into Qiagen® collection microtubes, and stored at −80 °C until further processing. DNA was extracted using a Qiagen® MagAttract® 96 DNA Plant Core Kit (Qiagen®, Hilden, Germany) following the manufacturer's protocol with minor modifications to enhance DNA recovery. Specifically, 33 µL of RB buffer and 10 µL of resuspended MagAttract suspension G were preloaded to each well of a 96-well microplate prior to extraction.

Amplicon library preparation for 16S rRNA sequencing on an Illumina MiSeq® system targeted the V4 hypervariable region, approximately 254 bp in length [4]. A two-step PCR amplification was performed. The first PCR utilised 515F and 806R primers, each tagged with Illumina adapter sequences. To minimise host DNA contamination, peptide nucleic acid (PNA) PCR blockers, pPNA and mPNA, were included to prevent the amplification of chloroplast and mitochondrial 16S rRNA genes [5]. The first PCR reaction (25 µL total volume) consisted of 12.5 µL Kapa HiFi HotStart 2× Ready Mix, 1.25 µL of 50 µM pPNA and mPNA mix, 1 µL of 5 µM forward and reverse primers, 5 µL of template DNA, and PCR-grade water. The thermal cycling conditions included an initial denaturation at 94 °C for 3 minutes (min), followed by 30 cycles of denaturation at 94 °C for 15 seconds (sec), PNA clamping at 75 °C for 10 sec, annealing at 55 °C for 10 sec, elongation at 72 °C for 45 sec, and a final extension at 72 °C for 10 min. Post-amplification, size-selective PCR purification was conducted using ProNex® magnetic beads at a 1:1.35 volumetric ratio (PCR product: ProNex® beads) to remove excess primers and unwanted fragments. A second index PCR was performed to attach Illumina sequencing adaptors and dual indices using a Nextera XT Index Kit (Illumina, San Diego, CA, USA). The 50 µL reaction contained 25 µL 2× KAPA HiFi HotStart Ready Mix, 5 µL of each Nextera XT index primer (N7 and S5), 5 µL of template DNA, and PCR-grade water. The thermal cycling conditions were: 95 °C for 3 min, followed by 8 cycles of 95 °C for 30 sec, 55 °C for 30 sec, 72°C for 30 sec, and a final extension at 72 °C for 5 min.

The amplified PCR products were validated using Agilent Tapestation 2200 (Agilent Technologies, Santa Clara, CA, USA) with High Sensitivity D1000 (HSD1000) Screentape assay to confirm fragment sizes. Purification and normalisation of amplicons were performed using the SequalPrep™ Normalisation Plate (Thermo Fisher Scientific, Waltham, MA, USA) according to the manufacturer's protocol. The libraries were pooled in equal volumes (5 µL per sample) and quantified using a NanoDrop™ 2000/2000c spectrophotometer (Thermo

Scientific™, Waltham, MA, USA) and a Quantus™ fluorometer (Promega, Madison, WI, USA) with QuantiFluor® dsDNA assay.

The final pooled library was denatured using 0.2N NaOH, diluted to 6 pM, and spiked with PhiX control (15% of final concentration) to improve sequencing diversity. According to the Illumina MiSeq protocol, 600 µL of the final library was loaded onto the MiSeq reagent cartridge, and sequencing was performed using a 2 × 300 bp MiSeq Reagent Kit v3 chemistry for paired-end sequencing.

Supplementary Section S5

Evaluating the effects of SynCom treatments on plant growth and biomass at 76 DAP under post-stress recovery conditions

Following re-establishment in the glasshouse after the PPV experiment, growth recovery responses were assessed at 76 DAP in lucerne plants previously subjected to either well-watered or drought conditions. Under well-watered conditions, the LA treatment recorded the highest mean root length (36.13 ± 2.15 cm), shoot length (51.07 ± 11.06 cm), and shoot biomass 26.06 ± 2.63 g), whereas the LT treatment showed the highest root biomass (33.25 ± 15.17 g). In contrast, among plants that had experienced drought, the Mix treatment showed the highest root length (35.77 ± 2.27 cm), and the LT treatment again recorded the highest root biomass (10.39 ± 11.03 g). However, shoot length and biomass were particularly lower across all the SynCom-treated groups compared to their Control plants previously subjected to drought (shoot length = 30.80 ± 4.38 cm; shoot biomass = 9.85 ± 4.83 g).

Statistical analysis confirmed normal data distribution (Shapiro-Wilk test, $p > 0.05$; Levene's test, $p > 0.05$), allowing the use of one-way ANOVA. A statistically significant difference was observed for shoot biomass across watering conditions (p value = 6.95E-09), but pairwise post-hoc Tukey's tests did not reveal any significant differences between SynCom treatments and their respective Controls based on prior watering regimes. Each SynCom treatment was compared to its corresponding uninoculated Control group that had previously been subjected to either well-watered or drought conditions at the PPV ($n = 3$). While some treatments showed elevated mean values, root biomass under LT displayed particularly high variability.

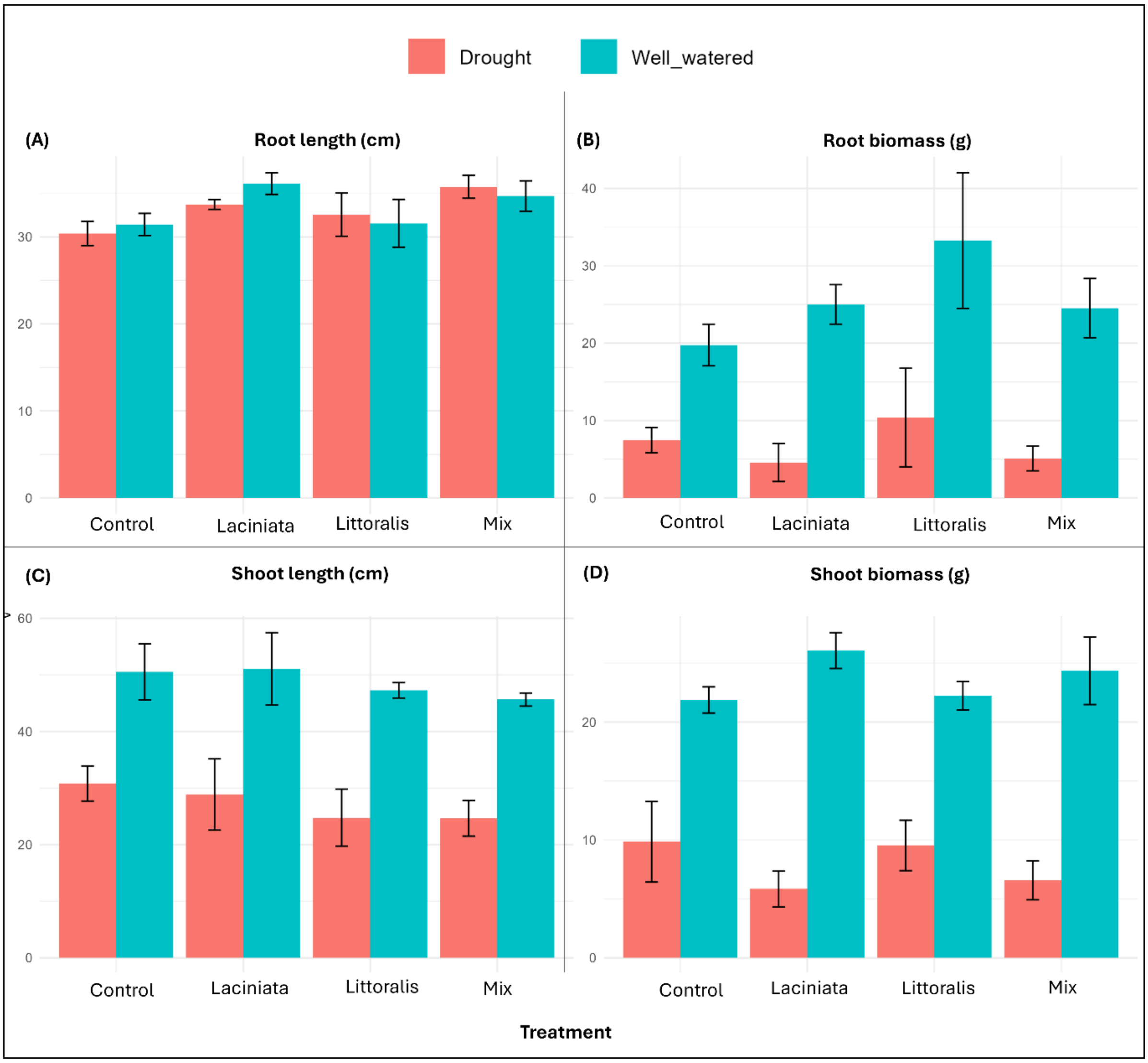

Supplementary Figure S2. Root and shoot growth responses of lucerne seedlings at 76 DAP under different SynCom treatments. (A) Root length, (B) root biomass, (C) shoot length, and (D) shoot biomass was measured in plants treated with Control and SynCom treatments: Laciniata, Littoralis, and Mix. Bars represent mean ± standard error (SE). Measurements indicate post-stress recovery dynamics following different SynCom inoculations.

Supplementary Section S6

<u>Comparison of growth parameters across time-points</u>

Comparative analysis of root and shoot measurements between 24 DAP and 76 DAP

A comparison of root and shoot fold changes between 24 DAP and 76 DAP revealed distinct biological trends across SynCom treatments. The LT treatment exhibited the highest root fold change (3.66), closely followed by LA (3.58), the Control (3.52) and the Mix treatment (2.81) (Figure S2A). For shoot fold changes, the Control group had the highest value (3.52), followed by LT (3.06), LA (3.04) and Mix treatment (2.45). Overall, the Mix treatments showed the lowest fold changes for both root and shoot growth. Fold change values were calculated as the ratio of mean measurements at 76 DAP relative to 24 DAP, based on unpaired group means from 10 replicates at 24 DAP and three replicates at 76 DAP. Statistical analysis using one-way ANOVA revealed no significant differences in fold change values among treatments for either root or shoot length ($F = 1.50$, $df = 3$, $p = 0.220$).

Statistical analysis confirmed normal data distribution (Shapiro-Wilk test, $p > 0.05$; Levene's test, $p > 0.05$), allowing the use of one-way ANOVA. A statistically significant difference was observed for shoot biomass across watering conditions (p value = 6.95E-09), but pairwise post-hoc Tukey's tests did not reveal any significant differences between SynCom treatments and their respective controls based on prior watering regimes. Each SynCom treatment was compared to its corresponding uninoculated Control group that had previously been subjected to either well-watered or drought conditions at the PPV ($n = 3$). While some treatments showed elevated mean values, root biomass under LT displayed particularly high variability.

Comparative analysis of aerial biomass measurements between 63 DAP and 76 DAP

A comparison of wet aerial biomass measurements between 63 DAP and 76 DAP revealed an overall increase in the aerial biomass across all treatment groups at 76 DAP. Fold change analysis demonstrated the highest increase in the LA treatment (2.11), followed by Mix (1.95), LT (1.62), and Control (1.20) (Figure S2B). Fold changes were calculated using group-level means from unpaired samples, with three replicates at 76 DAP and five replicates at 63 DAP. As biomass fold change data did not meet the assumptions of normality and homogeneity of variance, a non-parametric Kruskal-Wallis test was applied. No statistically significant differences were detected among the treatments ($p = 0.39$).

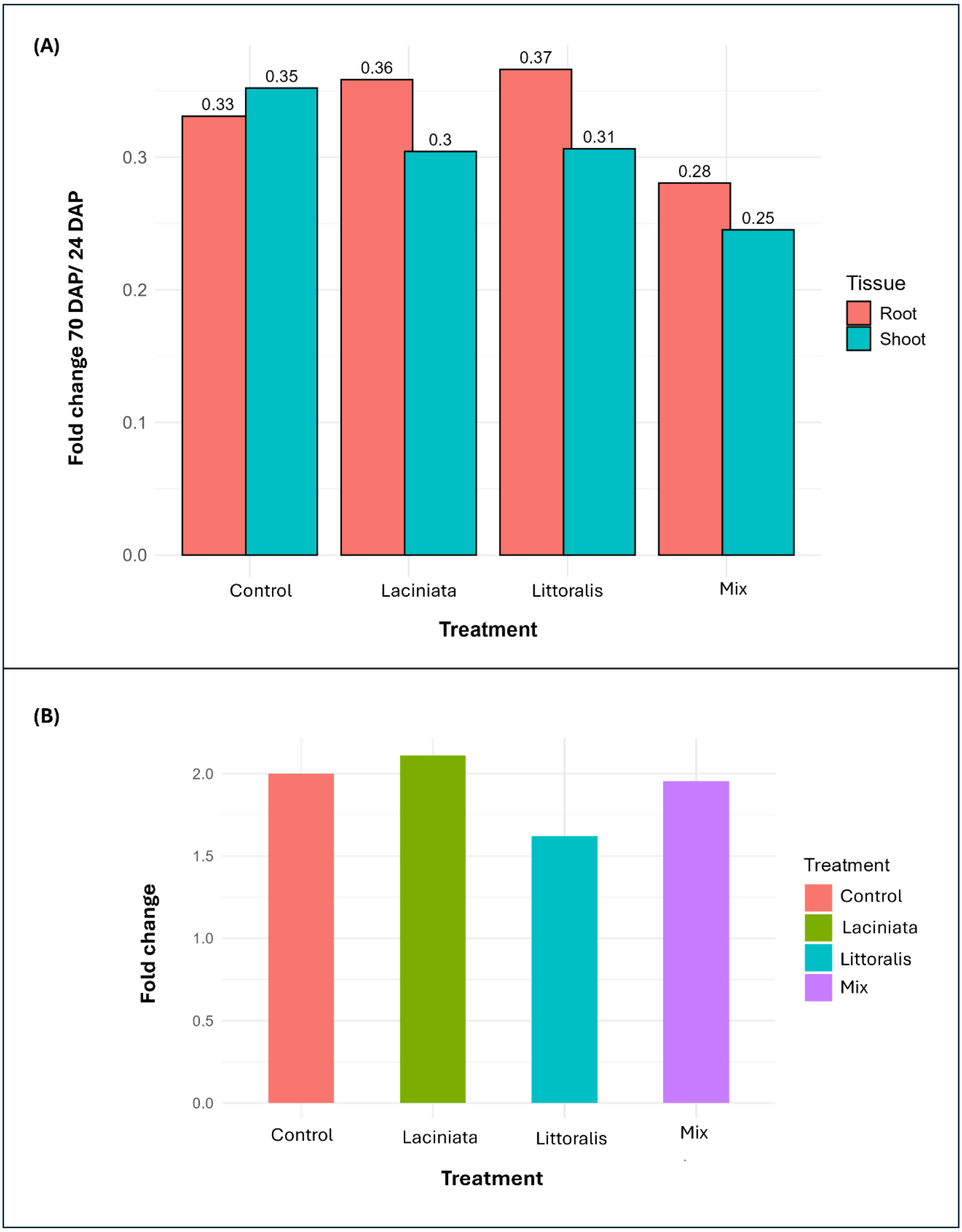

Supplementary Figure S3. Fold change analysis of plant growth across developmental stages. (A) Root and shoot length fold changes from 24 DAP and 76 DAP across Control, Laciniata, Littoralis, and Mix treatments. Fold changes were calculated as the ration of mean measurements at 76 DAP relative to 24 DAP. (B) Fold changes in wet aerial biomass between 63 DAP to 76 DAP across treatments. Bars represent mean fold change values.

Supplementary Section S7

SynCom performance during post-stress recovery and later growth stages

Following the exploratory phenotyping at 76 DAP (see Section 3.2.3), we examined potential treatment-specific trends in post-stress recovery. While replication was limited, the patterns observed offer preliminary insights into SynCom-associated growth trajectories under fluctuating conditions.

The transition from the PPV facility to glasshouse included a four-hour transport and acclimation period, which may have introduced mechanically induced stress (MIS), followed by adjustment to new watering and light regimes. Plants experiencing MIS often activate defence signalling pathways, including the accumulation of ascorbic acid and polyphenolic compounds, which contribute to stress adaptation and recovery [6]. These external factors may have influenced recovery outcomes, highlighting both plant resilience and the potential role of SynComs in facilitating post-stress adaptation.

At 76 DAP, conventional phenotyping indicated treatment-specific trends in post-stress recovery. LA-inoculated plants that were previously well-watered showed comparatively improved root and shoot traits, suggesting enhanced biomass recovery (Supplementary Section S2: Figure S1). In contrast, LT-treated plants exhibited a more root-centric growth strategy, potentially supporting long-term nutrient acquisition and physiological stability. This trend may relate to the early enrichment of Cytophagales, Chitinophagales, and Saccharimonadales observed at 24 DAP—microbial groups associated with nutrient cycling, hormone regulation, and root development [7-9].

Post-stress recovery is increasingly recognised as a relevant yet underexplored phenotype in plant–microbiome research, particularly for crops exposed to variable environmental conditions. In this study, SynCom treatments offered limited aboveground recovery in drought-affected plants. Notably, Control plants outperformed SynCom-treated groups in shoot biomass, aligning with previous findings by Yadav, *et al.* [10], who reported variable SynCom effects on recovery. However, root-level advantages were apparent in some treatments: Mix-inoculated plants exhibited the greatest root length, while LT-treated plants recorded the highest root biomass, potentially indicating an adaptive shift toward belowground investment during recovery.

The limited aboveground recovery observed in SynCom-treated drought plants may reflect a lack of specific functional traits, such as osmoprotectant synthesis, rapid reactivation under stress, or efficient water-use strategies required for effective post-stress resilience. These observations reinforce the importance of designing SynComs not only for initial colonisation or drought adaptation, but also for functional persistence during recovery. The comparatively reduced performance of Mix-treated plants, despite strong early-stage outcomes, could be attributed to functional redundancy, insufficient microbial activation under later growth conditions, or competitive suppression by native microbiota in a changing environment.

Microbiome insights from 24 DAP offer a possible mechanistic basis for these phenotypic patterns. LA-inoculated plants, which appeared to recover more robustly, had previously shown enrichment in *Azospirillum*, Sphingomonadales, and Burkholderiales—taxa widely associated with drought adaptation, osmoprotection, and microbiome stabilisation [11-13]. Although microbiome profiling was limited to 24 DAP, the phenotypic trends observed at 76 DAP suggest that early microbial signatures may have had lingering effects in some treatments (e.g., LA), even as community composition likely evolved. In contrast, the reduced biomass response in Mix-treated plants indicated that early benefits may not be sustained without continued compatibility or functional expression over time.

Comparative analysis of root and shoot fold changes between 24 DAP and 76 DAP suggested stage-dependent SynCom efficacy. LT-inoculated plants exhibited the greatest increase in root fold change (Supplementary Section 2: Figure S2), while Mix-treated plants showed the lowest fold changes for both root and shoot traits, consistent with a decline in SynCom efficacy at later developmental stages. Similar trends have been reported in cotton [14], where early SynCom benefits diminished over time, possibly due to host filtering or microbial competition with native soil microbiota [15]. As plants transition to reproductive phases, resource allocation often shifts away from vegetative growth, contributing to slower biomass accumulation [15,16]. Additionally, premature flowering has been associated with reduced vegetative growth, which may partially explain lower shoot biomass observed in later stages [17].

Further comparison of wet aerial biomass between 63 DAP (well-watered) and 76 DAP (recovery under normal watering) showed biomass increases across all treatments. Among SynCom-inoculated groups, the LA-treated plants exhibited the highest fold increase, further suggesting a modest advantage in recovery potential. While these findings remain exploratory due to small sample size, they highlight the potential of drought-adapted SynComs, such as those derived from *M. laciniata* (LA), to support resilience beyond the immediate recovery phase.

References

1. Gehan, M.A.; Fahlgren, N.; Abbasi, A.; Berry, J.C.; Callen, S.T.; Chavez, L.; Doust, A.N.; Feldman, M.J.; Gilbert, K.B.; Hodge, J.G.; et al. PlantCV v2: Image analysis software for high-throughput plant phenotyping. *PeerJ* **2017**, *5*, e4088, https://doi.org/10.7717/peerj.4088.
2. Dimech, A.M.; Kaur, S.; Breen, E.J. Mapping and quantifying unique branching structures in lentil (*Lens culinaris* Medik.). *Plant Methods* **2024**, *20*, 95, https://doi.org/10.1186/s13007-024-01223-1.
3. Klukas, C.; Chen, D.; Pape, J.-M. Integrated analysis platform: An open-source information system for high-throughput plant phenotyping *Plant Physiol.* **2014**, *165*, 506-518, https://doi.org/10.1104/pp.113.233932.
4. Hall, M.; Beiko, R.G. 16S rRNA gene analysis with QIIME2. In *Microbiome Analysis: Methods and Protocols*, Beiko, R.G., Hsiao, W., Parkinson, J., Eds.; Springer New York: New York, NY, USA, 2018; pp. 113–129.
5. Lundberg, D.S.; Yourstone, S.; Mieczkowski, P.; Jones, C.D.; Dangl, J.L. Practical innovations for high-throughput amplicon sequencing. *Nat. Methods* **2013**, *10*, 999–1002, https://doi.org/10.1038/nmeth.2634.
6. Šic Žlabur, J.; Radman, S.; Fabek Uher, S.; Opačić, N.; Benko, B.; Galić, A.; Samirić, P.; Voća, S. Plant response to mechanically-induced stress: A case study on specialized metabolites of leafy vegetables. *Plants* **2021**, *10*, 2650, https://doi.org/10.3390/plants10122650.
7. Ikeda, S.; Okazaki, K.; Takahashi, H.; Tsurumaru, H.; Minamisawa, K. Seasonal shifts in bacterial community structures in the lateral root of sugar beet grown in an andosol field in Japan. *Microbes Environ.* **2023**, *38*, ME22071, https://doi.org/10.1264/jsme2.ME22071.
8. Sun, C.; Xiao, J.; Bai, L.; Bai, J.; Liu, J.; Geng, L.; Zhang, Y. Defined and natural PAH contaminations shift PAH-degrading bacterial community in rhizosphere of ornamental plant species *Echinacea purpurea* L. *Environ. Technol. Innov.* **2023**, *31*, 103189, https://doi.org/10.1016/j.eti.2023.103189.

9. Kim, H.S.; Kim, J.-S.; Suh, M.K.; Eom, M.K.; Lee, J.; Lee, J.-S. A novel plant growth-promoting rhizobacterium, *Rhizosphaericola mali* gen. nov., sp. nov., isolated from healthy apple tree soil. *Sci. Rep.* **2024**, *14*, 1038, https://doi.org/10.1038/s41598-024-51492-y.
10. Yadav, A.; Chen, M.; Acharya, S.M.; Yang, Y.; Zhao, T.Z.; Chakraborty, R. A stable 15-member bacterial SynCom promotes *Brachypodium* growth under drought stress. *bioRxiv* **2024**, https://doi.org/10.1101/2024.09.10.612297.
11. Arzanesh, M.H.; Alikhani, H.A.; Khavazi, K.; Rahimian, H.A.; Miransari, M. Wheat (*Triticum aestivum* L.) growth enhancement by *Azospirillum* sp. under drought stress. *World J. Microbiol. Biotechnol.* **2011**, *27*, 197–205, https://doi.org/10.1007/s11274-010-0444-1.
12. Bano, Q.; Ilyas, N.; Bano, A.; Zafar, N.; Akram, A.; Hassan, F. Effect of *Azospirillum* inoculation on maize (*Zea mays* L.) under drought stress. *Pak J Bot* **2013**, *45(S1)*, 13–20.
13. Jha, Y.; Subramanian, R.B. Plant microbiome: Stress response. In *Interaction to Gene Induction: An Ecofriendly Mechanism of PGPR-mediated Stress Management in the Plant*, Egamberdieva, D., Ahmad, P., Eds.; Springer: Singapore, 2018; pp. 217–232.
14. Kaur, S.; Egidi, E.; Qiu, Z.; Macdonald, C.A.; Verma, J.P.; Trivedi, P.; Wang, J.; Liu, H.; Singh, B.K. Synthetic community improves crop performance and alters rhizosphere microbial communities. *J. Sustain. Agric. Environ.* **2022**, *1*, 118–131, https://doi.org/10.1002/sae2.12017.
15. Pereg, L.; McMillan, M. Scoping the potential uses of beneficial microorganisms for increasing productivity in cotton cropping systems. *Soil Biol. Biochem.* **2015**, *80*, 349–358, https://doi.org/10.1016/j.soilbio.2014.10.020.
16. Berg, G.; Rybakova, D.; Grube, M.; Köberl, M. The plant microbiome explored: Implications for experimental botany. *J. Exp. Bot.* **2015**, *67*, 995–1002, https://doi.org/10.1093/jxb/erv466.
17. Jiang, X.; Yang, T.; Zhang, F.; Yang, X.; Yang, C.; He, F.; Long, R.; Gao, T.; Jiang, Y.; Yang, Q.; et al. RAD-seq-based high-density linkage maps construction and quantitative trait loci mapping of flowering time trait in alfalfa (*Medicago sativa* L.). *Front. Plant Sci.* **2022**, *13*, 899681, https://doi.org/10.3389/fpls.2022.899681.

OFFICIAL
